# *In silico* selection of functionally important proteins from the mialome of *Ornithodoros erraticus* ticks and assessment of their protective efficacy as vaccine targets

**DOI:** 10.1186/s13071-019-3768-1

**Published:** 2019-10-30

**Authors:** Ricardo Pérez-Sánchez, Raúl Manzano-Román, Prosper Obolo-Mvoulouga, Ana Oleaga

**Affiliations:** 1 Parasitología Animal, Instituto de Recursos Naturales y Agrobiología de Salamanca (IRNASA, CSIC), Cordel de Merinas, 40–52, 37008 Salamanca, Spain; 2 Present Address: Proteomics Unit, Cancer Research Centre (IBMCC/CSIC/USAL/IBSAL), 37007 Salamanca, Spain

**Keywords:** *Ornithodoros erraticus*, Mialome, Vaccines, Chitinase, Ribosomal protein P0, Tetraspanin, Secreted protein PK-4

## Abstract

**Background:**

New candidate protective antigens for tick vaccine development may be identified by selecting and testing antigen candidates that play key biological functions. After blood-feeding, tick midgut overexpresses proteins that play essential functions in tick survival and disease transmission. Herein, *Ornithodoros erraticus* midgut transcriptomic and proteomic data were examined in order to select functionally significant antigens upregulated after feeding to be tested as vaccine candidate antigens.

**Methods:**

Transcripts annotated as chitinases, tetraspanins, ribosomal protein P0 and secreted proteins/peptides were mined from the recently published *O. erraticus* midgut transcriptome and filtered in a second selection step using criteria based on upregulation after feeding, predicted antigenicity and expression in the midgut proteome. Five theoretical candidate antigens were selected, obtained as recombinant proteins and used to immunise rabbits: one chitinase (CHI), two tetraspanins (TSPs), the ribosomal protein P0 (RPP0) and one secreted protein PK-4 (PK4).

**Results:**

Rabbit vaccination with individual recombinant candidates induced strong humoral responses that mainly reduced nymph moulting and female reproduction, providing 30.2% (CHI), 56% (TSPs), 57.5% (RPP0) and 57.8% (PK4) protection to *O. erraticus* infestations and 19.6% (CHI), 11.1% (TSPs), 0% (RPP0) and 8.1% (PK4) cross-protection to infestations by the African tick *Ornithodoros moubata.* The joint vaccine efficacy of the candidates was assessed in a second vaccine trial reaching 66.3% protection to *O. erraticus* and 25.6% cross-protection to *O. moubata*.

**Conclusions:**

These results (i) indicate that argasid chitinases and RPP0 are promising protective antigens, as has already been demonstrated for ixodid chitinases and RPP0, and could be included in vaccines targeting multiple tick species; (ii) reveal novel protective antigens tetraspanins and secreted protein PK-4, never tested before as protective antigens in ticks; and (iii) demonstrate that multi-antigenic vaccines increased vaccine efficacy compared with individual antigens. Lastly, our data emphasize the value of the tick midgut as a source of protective candidate antigens in argasids for tick control.

## Background

Tick infestations and tick-borne diseases are a growing threat for human and animal health globally [1]. *Ornithodoros erraticus* is an argasid tick species reported to be the main vector of tick-borne human relapsing fever (TBRF) and of African swine fever (ASF) in the Mediterranean Basin [2–4]. Moreover, *O. erraticus* is the type-species of the “*O. erraticus* complex”, and several species in this complex, including *O. asperus*, *O. lahorensis*, *O. tartakovsky* and *O. tholozani*, are distributed through the Middle East, the Caucasus, the Russian Federation and the Far East, where they transmit different species of TBRF-causing borreliae [5–7] and where the ASF virus has penetrated and spread out of control in the last decade [8–11]. Although this has not been experimentally proven hitherto, if these tick species in the “*O. erraticus* complex” were also competent vectors of the ASF virus, their presence in anthropic environments would significantly increase the transmission and long-term persistence of ASF in this wide region. Accordingly, the effective prevention and control of TBRF and ASF would necessarily go through eradicating the *Ornithodoros* vectors from at least anthropic environments [12]. While chemical acaricide agents are not effective against these *Ornithodoros* ticks [12], alternative methods for the control of ticks are urgently required and tick vaccines have been validated as an effective sustainable method for the control of tick infestations and tick-borne diseases [13–15].

The tick midgut is an essential organ for tick survival because it manages host blood digestion and the absorption of the released nutrients, contributes to protection against host immunity and participates in blood-borne pathogen infection and transmission [16–19]. Accordingly, midgut proteins involved in these processes may be interesting targets for developing vaccines aimed to control ticks and tick-borne pathogens. In fact, in our previous studies with *Ornithodoros* ticks we have observed that plasma membrane-associated proteins from tick enterocytes induce protective immune responses in vaccinated animals [20–22].

The midgut transcriptomes and proteomes (mialomes) of *O. erraticus* females, taken before feeding and 48 hours post-feeding, have recently been obtained providing a wealth of information on the physiology of blood digestion and the functionally relevant proteins that are upregulated in the *O. erraticus* midgut in response to blood feeding (BioProject PRJNA377416) [23, 24].

Some of these proteins, including two aquaporins, one ATP-binding cassette (ABC) transporter and one selenoprotein T (OeSEL), have recently been selected and tested as protective candidate antigens, obtaining partial protection against *O. erraticus* infestations, which reached 47.5% vaccine efficacy for OeSEL [25]. Thus, new candidate protective antigens from *Ornithodoros* ticks are still needed, which may be identified by searching the *O. erraticus* mialome for proteins playing a role in physiological processes that are essential for tick survival.

Accordingly, in the present study, we have focused on midgut chitinases, tetraspanins and the 60S acidic ribosomal protein P0 (RPP0) for the selection and testing of new candidate protective antigens.

Chitinases are enzymes that hydrolyse the β-1,4-glycosidic bond between *N*-acetyl-d-glucosamine moieties, mainly found in chitin. In arthropods, chitin is a major structural component of the exoskeleton and an essential part of the peritrophic matrix lining the gut epithelium. The peritrophic matrix is a permeability barrier that protects the midgut from mechanical damage, toxins and pathogens. In order to grow and develop, arthropods need to remodel their chitin-containing structures, which requires the involvement of chitinases and chitin synthases [26]. Arthropod chitinases belong to the glycoside hydrolase-18 (GH18) family, which includes numerous and diverse enzymes with a modular structure composed of a combination of catalytic domains (GH18 domains), cysteine-rich chitin-binding domains, and serine/threonine-rich linker domains. These chitinases are differently expressed between developmental stages and tissues and play distinct functions [27]. In ticks, chitinases have been found in salivary glands, the midgut, ovaries, Malpighian tubules, synganglion and the epidermis, where they contribute to attaching and feeding, degradation of the chitinous endocuticle during moulting, and regulation of the turnover and porosity of the chitin-containing peritrophic matrix [24, 28–30]. Accordingly, tick chitinases have been considered as potential bioacaricides and vaccine targets for tick control [28, 31]. More recently, RNAi gene knockdown of a chitinase of *Amblyomma americanum* was shown to reduce female feeding performance and fecundity [29].

Tetraspanins are evolutionarily conserved membrane proteins that tend to associate with one another and to cluster dynamically with numerous and varied partner proteins forming tetraspanin-enriched microdomains in the cell membrane. Thus, tetraspanins are involved in the coordination of numerous intracellular and intercellular biological processes including signalling, cell proliferation, adhesion and migration, cell fusion, immune defence and host-parasite interactions [32–34]. Increasing evidence shows that host tetraspanins (vertebrate and invertebrate) are exploited by viruses, bacteria, protozoa and pathogenic fungi for infection, dissemination and transmission [35–39]. Conversely, endogenous tetraspanins of lower parasitic eukaryotes, including schistosomes and filarial nematodes, are often pivotal for infectivity and survival in their hosts [33]. Due to their roles in pathogen-vector-host interactions, tetraspanins are potential targets for new therapies and vaccines aimed at controlling parasite infections, arthropod vectors and vector-borne diseases. Actually, a tetraspanin-based candidate vaccine to prevent human schistosomiasis is currently under development [33, 40]. Regarding ticks, tetraspanins might also be suitable targets for vaccines aimed at controlling tick infestations and tick-transmitted pathogens, but no studies on the protective efficacy of tick tetraspanins have been hitherto undertaken.

The 60S acidic ribosomal protein P0 (RPP0) is a highly conserved multifunctional protein in eukaryotes. It is a structural component of ribosomes involved in protein synthesis and its absence results in defective 60S ribosomal subunits, which are inactive for protein synthesis, and cell death [41–43]. Additionally, a regulatory role of RPP0 in DNA repair, apoptosis, cell development and carcinogenesis has also been documented [44]. RPP0 has also been shown to associate to membrane proteins on the cell surface of yeast, apicomplexan parasites and mammalian cell lines, but its function there still is unknown [45]. RPP0 has proven to be immunogenic and a promising vaccine candidate against several parasitic protozoans [42], and it has also been explored as a protective candidate antigen in ixodid ticks. RNAi gene knockdown of RPP0 in *Haemaphysalis longicornis* resulted in a dramatic reduction in tick feeding and a mortality of 96%, suggesting that RPP0 is essential for blood ingestion and tick viability [41]. More recently, rabbit vaccination with a synthetic peptide from the RPP0 of *Rhipicephalus sanguineus* showed 90% vaccine efficacy against this species, and cattle vaccination with this same peptide provided 96% vaccine efficacy against *Rhipicephalus microplus* infestations [42, 43].

Besides these proteins, we were also interested in a group of transcripts from the *O. erraticus* mialome annotated as “secreted protein” and “secreted peptide”. Despite most of them have unknown functions, they may be interesting as candidate antigens because secreted antigens are easily reached by host antibodies in the tick midgut lumen.

All the above-mentioned observations highlight these tick proteins as potential protective antigens and prompted us to study their vaccine efficacy. Thus, in the present study, one chitinase, two tetraspanins, the ribosomal protein P0 and one secreted protein PK-4 were selected from the *O. erraticus* mialome, and their individual and joint vaccine efficacy tested to *O. erraticus* and the African *Ornithodoros moubata* soft ticks.

## Methods

### Ticks and tick material

The *O. erraticus* and *O. moubata* ticks used in this study came from the laboratory colonies kept at IRNASA (CSIC), Spain. The *O. erraticus* colony originated from specimens captured in Sanchón de la Ribera (41°5ʹ0″N, 6°25ʹ0″W), Salamanca Province, western Spain. The *O. moubata* colony was started from specimens kindly provided by the Institute for Animal Health in Pirbright (Surrey, UK). The ticks are regularly fed on rabbits (New Zealand white) and maintained at 28 °C, 85% relative humidity and a 12:12 h L:D photoperiod.

Midguts from engorged *O. erraticus* females 48 h post-feeding (hpf) were obtained as described by [24] and preserved in RNA-later (Ambion, Austin, USA) for RNA extraction. Total RNA was extracted and purified using the RNeasy Mini Kit (Qiagen, Hilden, Germany).

Additionally, midgut tissues obtained from both *O. erraticus* and *O. moubata* unfed females and fed females 48 hpf were used to prepare protein extracts enriched in either soluble or membrane-associated proteins [23]. Briefly, batches of 25 midguts from each species and physiological condition were homogenised and sonicated in ice-cold phosphate-buffered saline (PBS) (pH 7.4) supplemented with proteinase inhibitors (Roche Diagnostics, Indianapolis, USA). The homogenates were centrifuged at 10^4^×*g* and the 10^4^ g supernatants, free of particulate material, were re-centrifuged at 10^5^×*g* into new supernatants and pellets containing, respectively, the soluble and membrane proteins. Protein concentration in the midgut extracts was assessed with the BCA Protein Assay Reagent kit (Thermo Fisher Scientific, Rockford, USA). The extracts were stored at – 20 °C until use.

Tick saliva was collected from *O. erraticus* and *O. moubata* females following the protocol described by [46], that involved salivation stimulation with 1% pilocarpine. Protein concentration in the saliva samples was measured using the Bradford assay (Bio-Rad, Hercules, USA) and the samples were stored at – 20 °C.

### Analysis and selection of chitinases, tetraspanins, acidic ribosomal protein P0 and secreted proteins/peptides

Transcriptomic data regarding chitinases, tetraspanins, acidic ribosomal protein P0 and secreted proteins/peptides of *O. erraticus*, namely the transcript sequences and the transcription levels expressed in fragments per kilobase of transcript per million mapped reads (FPKM), were acquired from the *O. erraticus* midgut transcriptome, which has recently been obtained by our team [24]. The immunogenicity of the encoded proteins was predicted with the VaxiJen 2.0 software (http://www.ddg-pharmfac.net/vaxijen/vaxijen/vaxijen.html) using the 0.5 antigenicity threshold established by default for parasites [47–49].

One or two members from each of the above-mentioned protein families were chosen as potential protective candidate antigens. For this selection, proteins with the highest expression level, the highest fold-change after feeding and the highest predicted antigenicity (Vaxijen score) were prioritised. Additionally, the presence of these proteins in the *O. erraticus* midgut proteome [23] was also scored. For every selected candidate protein, its orthologues in argasid and ixodid ticks were searched in the Uniprot and NCBInr databases by BLASTp. The Clustal Omega alignment tool (https://www.ebi.ac.uk/Tools/msa/clustalo/) was used to align multiple orthologous amino acid sequences and identify conserved protein regions. Phylogenetic analyses of the aligned proteins were performed using the MEGA v.6 package [50]. Phylogenetic trees were built using the neighbour-joining method, gaps were treated as pairwise deletions, amino acid distances were calculated using the Poisson model, and branch supports were assessed by bootstrap analysis (10,000 bootstraps).

Topographical predictions of the amino acid sequence of every selected candidate, including their transmembrane and extracellular exposed regions, were analysed with the TMHMM and SACS TMHMM software (http://www.sacs.ucsf.edu/cgi-bin/tmhmm.py) [51, 52]. Additionally, the presence/absence of signal peptides, non-classical secretion signals and GPI anchors was checked using the SignalP-5.0 server (http://www.cbs.dtu.dk/services/SignalP/), the SecretomeP 2.0 server (http://www.cbs.dtu.dk/services/SecretomeP/) [53], and the GPI-SOM server (http://gpi.unibe.ch/) [54], respectively.

Prediction of secondary structures and three-dimensional (3D) modelling of the candidate proteins were done at the Phyre^2^ server [55]. The resulting 3D models were visualised using the Pymol package [56].

The presence of continuous linear B-cell epitopes on the selected candidate proteins was assessed using the following prediction tools: ABCpred (http://www.imtech.res.in/raghava/abcpred/index.html) [57], BCEpred (http://www.imtech.res.in/raghava/bcepred/) [58] and BepiPred-2.0 (http://www.cbs.dtu.dk/services/BepiPred/) [59]. The overlapping amino acid sequences in B-cell epitopes predicted by at least two of these tools were defined as the consensus predicted epitopes.

### Cloning of the candidates and production as recombinant proteins

The cDNA sequences encoding the full-length candidate OeRPP0, a truncated version (without signal peptide) of candidate OePK4, and the extracellular exposed regions of candidates OeCHI, OeTSP1 and OeTSP2 were cloned and expressed as recombinant proteins.

With this aim, the corresponding cDNA coding sequences were amplified by RT-PCR from total RNA from the midgut. Table 1 shows the PCR conditions for these amplifications and the specific primer pairs designed *ad-hoc*, which included suitable restriction sites to assist with subcloning into the pQE-30 (Qiagen) or pGEX-4T1 (GE Healthcare, Chicago, USA) expression vectors. The PCR products were first cloned in the pSC-A vector (Stratagene, LaJolla, USA) to verify their sequences. After that, they were digested and subcloned into the corresponding expression vector following previously described standard procedures [60, 61].

**Table 1:** Primers and PCR conditions used for amplification of the cDNA fragments encoding the target protein regions

Target protein	Target region (aa)	Primer name	Sequence (5ʹ-3ʹ)	Product size (bp)	Restriction enzymes	Expression vector
OeCHI	Extracellular domain (23–467)	Oe6961_BamH1_F	GGATCCCAGGACGGCGCCGCA	1350	*BamH1*; *Sma1*	pGEX-4T-1
		Oe6961_Sma1_	CCCGGGCTAGCACATGCCATTGATGCATTTAT			
OeTSP1*	Long extracellular domain (107–195)	Oe1520_BamH1_F	GGATCCAAGGTGGCTGACGGAGACA	282	*BamH1*; *Sma1*	pGEX-4T-1
		Oe1520_Sma1_R	CCCGGGCTAGACAGCACCCGTTCTCTCC			
OeTSP2	Long extracellular domain (124–213)	Oe8446_BamH1_F	GGATCCGCCTACGTCTCAACGTCCA	285	*BamH1*; *Sma1*	pQE-30
		Oe8446_Sma1_R	CCCGGGCTATGAGAGTCCATTGGATCGCA			
OePK4	Truncated, without signal P (22–109)	tOe9280_BamH1_F	GGATCC AGGCCTACCGAATCCGAGT	279	*BamH1*; *Sma1*	pQE-30
		Oe9280_Sma1_R	CCCGGGTTACTGGAGAGGGATCTCTAC			
OeRPP0	Whole protein (1–319)	OeRPP0_BamH1_F	GGATCCATGGTCAGGGAGGATAAGACA	972	*BamH1*; *Hind3*	pQE-30
		OeRPP0_Hind3_R	AAGCTTCTAATCAAAAAGTCCGAAGCCCA			

*Notes*: Primers include suitable restriction sites (underlined) to assist in the sub-cloning into the corresponding expression vector (last column)

PCR conditions were the same for all targets (94 °C 3 min; 10 × (94 °C for 15 s 59.5 °C for 30 s, 72 °C for 40 s) + 25 × (94 °C for 15 s, 66 °C for 30 s; 72 °C for 40 s); 72 °C for 7 min), except for TSP1, which required 60.5 °C in the first 10 amplification rounds

Recombinant pQE-30 plasmids containing the cDNA fragments coding for OePK4, OeRPP0 and OeTSP2 were transformed into *E. coli* M15 cells (Qiagen) and protein expression was induced with 1 mM IPTG following standard procedures. All of these proteins were expressed in 100% insoluble form. Thus, they were solubilised with 8 M urea, purified by nickel affinity chromatography in denaturing conditions, and dialysed against PBS (pH 7.4), for 24 h at 4 °C according to the procedure described by [61].

Recombinant pGEX-4T-1 plasmids containing the cDNA fragments coding for OeCHI and OeTSP1 were transformed into *Escherichia coli* BL21 cells and protein expression was induced with 0.1 mM IPTG. The CHI-GST and TSP1-GST fusion proteins were expressed in the 100% insoluble form. Therefore, they were solubilised with 8 M urea from the cellular lysate pellet and purified by electroelution from SDS-PAGE gels following the procedure described by [62]. The purified recombinant proteins were checked by SDS-PAGE and their identity was confirmed by in-gel enzymatic digestion followed by liquid chromatography and tandem mass spectrometry (LC-MS/MS) in a similar way as described in [23]. The concentration of the purified proteins was assessed by band densitometry in Coomassie blue-stained polyacrylamide gels and interpolation into a bovine serum albumin (BSA) standard curve. Purified proteins were stored at – 20 °C.

### Vaccine trial 1

The aim of this trial was to assess the capacity of the candidate recombinant antigens to induce protective immune responses in rabbits against infestations by *O. erraticus* and *O. moubata* ticks.

Except candidates TSP1-GST and TSP2, which were formulated together, each candidate antigen was individually formulated in Montanide ISA 50 V2 (Seppic, La Garenne Colombe Cedex, France) and administered to a group of three New Zealand white rabbits. Additionally, two groups of rabbits were included as controls: one group was treated with recombinant GST from *Schistosoma japonicum* (SjGST; Sigma-Aldrich, Saint Louis, USA) formulated in Montanide ISA 50 V2 and the other group was treated with the adjuvant alone. Each animal received three doses of 100 µg of the corresponding recombinant antigen administered subcutaneously at 15-day intervals.

Rabbits were bled immediately before administration of the first antigen dose (pre-immune sera), 14 days post-immunisation and immediately before tick infestation (14 dpi sera), and 14 days after the infestation (28 dpi sera). Blood samples were allowed to clot and sera were removed and stored at – 80 °C.

In the immune sera, their antibody titre to the homologous antigen and their reactivity to the other antigens were checked in ELISA following standard procedures [63]. Briefly, the ELISA plates were coated with 100 ng/well of recombinant antigen in 100 µl/well of carbonate buffer (pH 9.6), at 4 °C overnight, and post-coated with 1% BSA in PBS for 1 h at 37 °C. The sera were diluted in TPBS (PBS supplemented with 0.05% Tween 20) in a two-fold dilution series starting at 1/100, and each dilution was incubated in duplicate wells at 37 °C for 1 h. Peroxidase-conjugated anti-rabbit IgG (Sigma-Aldrich) was diluted 1/10,000 in TPBS and incubated at 37 °C for 1 h. Ortho-phenylene-diamine (OPD) (Sigma-Aldrich) was used as a chromogen substrate and the reactions were stopped with 3N sulphuric acid. The highest dilution of the immune serum giving more than twice the reactivity of the corresponding pre-immune serum at the same dilution was taken as the serum titre.

Once titrated, the immune sera were reacted in ELISA and western blot to tick saliva and the four midgut protein extracts (soluble and membrane proteins from fed and unfed females) from *O. erraticus* and *O. moubata* [22]. The ELISA plates were coated with 1 µg of saliva or midgut extract per well, the sera were diluted 1/300 in TPBS and the PO-anti-rabbit IgG was diluted 1/10,000.

Fourteen days after the last antigen dose, batches of 15 females, 30 males and 50 nymphs-3 of *O. erraticus,* and 15 females, 30 males and 50 nymphs-3 of *O. moubata* were allowed to feed on every rabbit for a maximum of 2 h. After that time, any tick still remaining on the animal was removed. To estimate the degree of protection, the following parameters were measured: the amount of blood ingested by every developmental stage analysed; the female oviposition and fertility rates (respectively, the number of eggs laid per female and newly hatched larvae/nymphs-1 per female); the moulting rate of nymphs-3; and the mortality rates of all the developmental stages tested.

The obtained data were subjected to statistical analysis using SPSS Statistics v25 software (IBM, Armonk, USA). For every parameter, the obtained values in the parasites that fed on each group of animals were summarised as the mean ± standard deviation. The overall differences among groups were compared by a one way-ANOVA. When global differences were detected in this analysis, a *post-hoc* test (bilateral Dunnett’s T-test) was applied to compare each vaccinated group to the merged control groups (adjuvant and SjGST) treated as a single control group. All statistical analyses were considered significant at the *p* < 0.05 level.

For each antigen formulation, the vaccine efficacy (E) was calculated according to the formula established by [64], and later updated by [65, 66]; this is based on comparing the reduction in the studied developmental processes between ticks fed on vaccinated animals and ticks fed on controls. Here, vaccine efficacy was calculated as E = 100 × [1 − (S × F × N × M)], where S and F are the reduction in the survival and fertility of female ticks, respectively, and N and M represent the reduction in survival and moulting of nymphs-3, respectively.

### Vaccine trial 2

The goal of this trial was to assess the combined vaccine efficacy of the more protective candidate antigens tested in the trial 1 (OeTSP1, OeTSP2, OeRPP0, OePK4) and the synthetic immunogenic peptide OeSEL, derived from the *O. erraticus* selenoprotein T, which was the more protective candidate in our preceding study [25].

For this, multi-antigenic formulation doses containing 100 μg of each candidate antigen in 1 ml of PBS were emulsified in an equal volume of Montanide ISA 50 V2 and administered to a group of three rabbits, following the same procedures as previously described for trial 1 for the assessment of cocktail efficacy. One additional group of rabbits was treated with Montanide ISA 50 V2 alone and used as the control.

## Results

### Chitinases: selected candidate OeCHI

Thirty-two transcripts annotated as chitinase and/or glycoside hydrolase-18 (GH-18 family) were recovered from the *O. erraticus* midgut transcriptome. Eleven of these transcripts were upregulated upon feeding (fold-change > 2) and five of them were also predicted to be antigenic (Vaxijen score > 0.5). Among the latter, transcript ci|000016961 showed by far the highest expression levels in the tick midgut, both before and after feeding (Additional file 1: Table S1). This transcript encodes a 492 amino acid long protein, which was also detected in the *O. erraticus* midgut proteome [23]. This transcript/protein was selected as a candidate antigen and termed OeCHI.

Uniprot and NCBInr databases searching for tick orthologues of OeCHI retrieved 12 top matches, comprising 3 argasid and 9 ixodid chitinases belonging to the GH-18 family. All of them showed E-values < 10^−70^ and amino acid sequence identities among 35% and 41% with sequence coverage between 64% and 78%. The amino acid sequence alignment of these 12 chitinases and OeCHI showed some conservation along the GH18 catalytic domain (amino acids 35–398 in OeCHI), which typically comprises four signature amino acid sequence motifs in arthropod chitinases, including the active site “FDG(L/F)DLDWE(Y/F)P” [27]. Outside the CH18 domain, the OeCHI showed no sequence conservation (Additional file 2: Figure S1a).

**Fig. 1:**
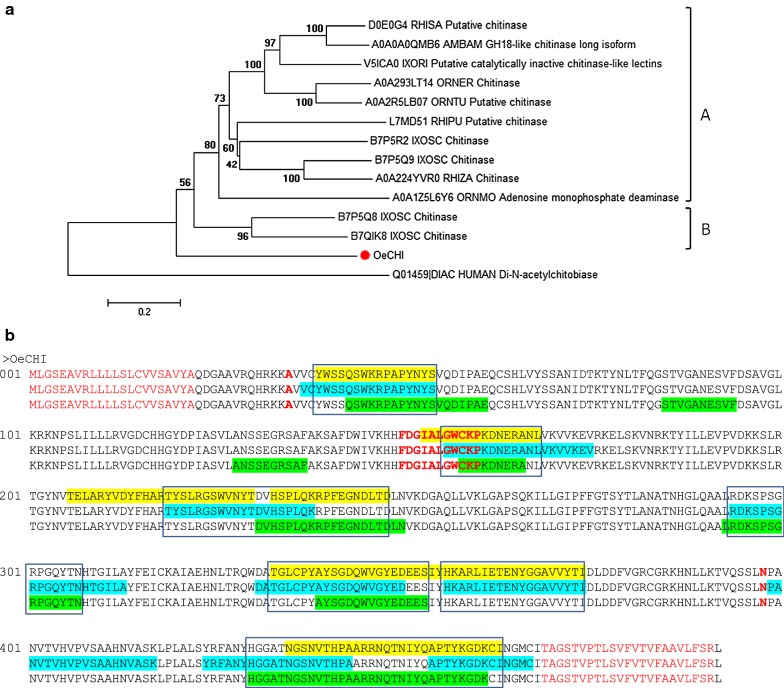
**a** Neighbour-joining analysis of the phylogenetic relationship of tick chitinases belonging to the glycoside hydrolase-18 (GH-18) family. OeCHI, *Ornithodoros erraticus* chitinase (red dot). Uniprot entry names are shown for the other sequences: AMBAM, *Amblyomma americanum*; IXORI, *Ixodes ricinus*; IXOSC, *I. scapularis*; ORNER, *Ornithodoros erraticus*; ORNMO, *O. moubata*; ORNTU, *O. turicata*; RHIPU, *Rhipicephalus pulchellus*; RHISA, *R. sanguineus*; RHIZA, *R. zambeziensis.* The *Homo sapiens* GH-18 chitobiase (Uniprot: Q01459) was included as outgroup reference. Evolutionary distances were computed using the Poisson correction method. Branch support values (10,000 bootstraps) for the nodes are indicated. **b** Linear B-cell epitope predictions for OeTSPs. The sequence of the protein is represented in triplicate showing the ABCpred (yelow), BCEpred (blue) and BepiPred-2.0 (green) predictions. The amino acids in epitopes predicted by at least two algorithms are highlighted in boxes. The signal peptide and transmembrane domain are depicted in red. The alanine 35 and asparagine 398 at the starting and end point of the GH-18 catalytic domain, respectively, and the active site FDGIALGWCKP are highlighted in bold red

The phylogenetic analysis of these tick GH18 chitinases clustered them into two main clusters, A and B, supported by 80% and 96% bootstrap values, respectively, whereas the OeCHI remained outside these clusters (Fig. 1a).

Topology prediction for OeCHI showed a single spanning transmembrane protein with a 22 amino acid long signal peptide, a large extracellular domain (aa 23–467) that contains the GH18 catalytic domain, a carboxy-terminal transmembrane domain (aa 468–490) and a very short cytoplasmic tail (aa 491–492) (Additional file 2: Figure S1b).

Linear B-cell epitope predictions for OeCHI are shown in Fig. 1b. Each immunoinformatics tool predicted a set of distinct but overlapping linear B-cell epitopes. Up to 7 epitopes were predicted by two or three algorithms, and their overlapping sequences were considered as the final predicted linear B-cell epitopes. Six of these epitopes were distributed throughout the GH-18 catalytic domain, actually covering the active site (Fig. 1b).

The three-dimensional (3D) modelling of OeCHI modelled up to 352 residues (72%) of its amino acid sequence with 100% confidence by the single highest scoring 3D template. The resulting 3D model showed the typical 3D structure for the catalytic region of GH-18 chitinases [67], i.e. the (α/β)8-TIM-barrel, which comprises 8 parallel β sheets forming a barrel in turn surrounded by 8 *α* helices that form a ring towards the outside (Additional file 2: Figure S1c). The six linear B-cell epitopes predicted for the GH-18 domain were located on the surface of OeCHI 3D model, where they could be easily accessed by host antibodies (Additional file 2: Figure S1c). The seventh predicted epitope could not be localised on the 3D model because it was close to the carboxy-end, outside the modelled region. For this protein, the whole extracellular region of OeCHI (aa 23–467) was cloned and produced as a recombinant candidate antigen (see below).

### Tetraspanins: selected candidates OeTSP1 and OeTSP2

Seventeen transcripts annotated as Tetraspanin (TM4SF) family members were recovered from the *O. erraticus* midgut transcriptome, but only seven were full length tetraspanins (200–350 amino acid residues) displaying the characteristic four transmembrane domains [32, 34]. All of them showed fold-changes after feeding between − 2.1 and 2.4, indicating that they were slightly or not differentially expressed upon feeding. Among them, four were predicted to be antigenic (Vaxijen scores > 0.5) and the two most antigenic also showed the highest expression levels in tick midgut both before and after feeding: namely, transcripts ci|000077740 (Uniprot: A0A293MYE4) and ci|000018446 (Uniprot: A0A293M0B7) (Additional file 1: Table S1). These transcripts encoded two proteins of 226 and 246 amino acid long, respectively, which were actually detected in the *O. erraticus* midgut proteome [23]. Hence, we selected both of them as candidate antigens and termed them OeTSP1 and OeTSP2, respectively.

Searching Uniprot and NCBInr databases for tick orthologues of OeTSP1 retrieved 12 tick sequences; eight of them were full-length, highly conserved argasid and ixodid tetraspanins, with E-values < 10^−120^ and more than 85% sequence identity (Additional file 3: Figure S2a). BLASTp searching for tick orthologues of OeTSP2 retrieved 10 tick sequences including full-length and fragment tetraspanins, which displayed E-values < 10^−33^ and a sequence identity between 30% and 58% (Additional file 3: Figure S2b). OeTSP1 and OeTSP2 had 27.3% sequence identity with each other (Additional file 3: Figure S2c).

**Fig. 2:**
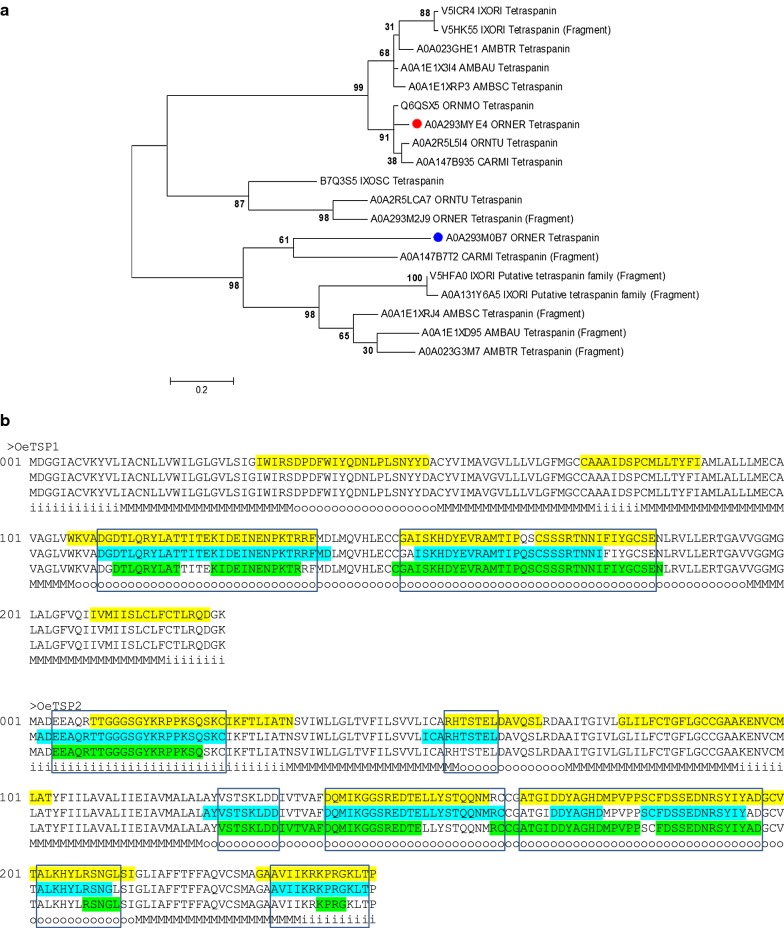
**a** Neighbour-joining analysis of the phylogenetic relationship of A0A293MYE4_ORNER (OeTSP1, red dot) and A0A293M0B7_ORNER (OeTSP2, blue dot) with their tick orthologues. Uniprot entry names are shown: AMBAU, *Amblyomma aureolatum*; AMBSC, *A. sculptum*; AMBTR, *A. triste*; CARMI, *Carios mimon*; IXORI, *Ixodes ricinus*; IXOSC, *I. scapularis*; ORNER, *Ornithodoros erraticus*; ORNMO, *O. moubata*; ORNTU, *O. turicata*. Evolutionary distances were computed using the Poisson correction method. Branch support values (10,000 bootstraps) for the nodes are indicated. **b** Linear B-cell epitope predictions for OeTSPs. The sequences of the proteins are represented in triplicate showing the ABCpred (yellow), BCEpred (blue) and BepiPred-2.0 (green) predictions. Epitopes predicted by at least two algorithms were highlighted in boxes. The predicted topology is indicated below the protein sequences to show that most epitopes mapped on the long extracellular domain: o (outside), extracellular; M, transmembrane; i, intracellular

The phylogenetic analysis of these TSPs showed a very close relationship between OeTSP1 and its orthologues, grouping them into one tight cluster supported by a 99% bootstrap value. The same analysis placed OeTSP2 and its orthologues into two different clusters, supported by 87% and 98% bootstrap values, which were poorly related to the OeTSP1 cluster (Fig. 2a).

Topological prediction for OeTSP1 and OeTSP2 confirmed that both are typical four-transmembrane tetraspanins with the characteristic short (13–18 amino acids) and long (88–89 amino acids) extracellular loops (Additional file 3: Figure S2d).

Linear B-cell epitope predictions are shown in Fig. 2b. For OeTSP1, the three algorithms predicted two large epitopes of 29 and 36 amino acids each that cover most of the sequence of the long extracellular loop, while the rest of the protein had no significant predictions. For OeTSP2, the three algorithms predicted four epitopes of 8, 24, 32 and 11 amino acids, covering almost the entire length of its long extracellular loop. Additionally, they also predicted two more epitopes on the cytoplasmic N- and C-termini, respectively.

3D modelling of OeTSP1 and OeTSP2 modelled up to 220 and 219 residues (97% and 89%), respectively, of their amino acid sequences with 100% confidence by the single highest scoring 3D template. The resulting 3D models displayed the typical 3D structure for monomeric Tetraspanin (TM4SF) family members, and showed the predicted epitopes on the surface of the long extracellular loops of both molecules, where they could be easily accessed by host antibodies (Additional file 3: Figure S2e). Hence, the two long extracellular loops were cloned and produced as recombinant candidate antigens (see below).

### Acidic ribosomal protein P0 (RPP0): candidate OeRPP0

Two transcripts annotated as the 60S acidic ribosomal protein P0 were recovered from the *O. erraticus* midgut transcriptome (Additional file 1: Table S1), but only one of them (ci|000113905) was upregulated upon feeding (fold-change, 2.78) and encoded a full-length ribosomal protein P0, namely, a 319 amino acid long protein without signal peptide, non-classical secretion signals, transmembrane domains or GPI anchors. This protein, termed OeRPP0, was also detected in the *O. erraticus* midgut proteome [23] and was selected as a vaccine candidate despite its predicted antigenicity being below the threshold (VaxiJen score 0.444).

Uniprot and NCBInr databases searching for tick orthologues of OeRPP0 retrieved 20 highly conserved tick sequences, all of them with an E-value = 0 and more than 90% sequence identity. Multiple alignment of these proteins confirmed their high conservation degree, including the 8 amino acid residues that form the 28S rRNA interface and the 20 residues that constitute the putative interface with the L7/L12 ribosomal proteins (Additional file 4: Figure S3a).

**Fig. 3:**
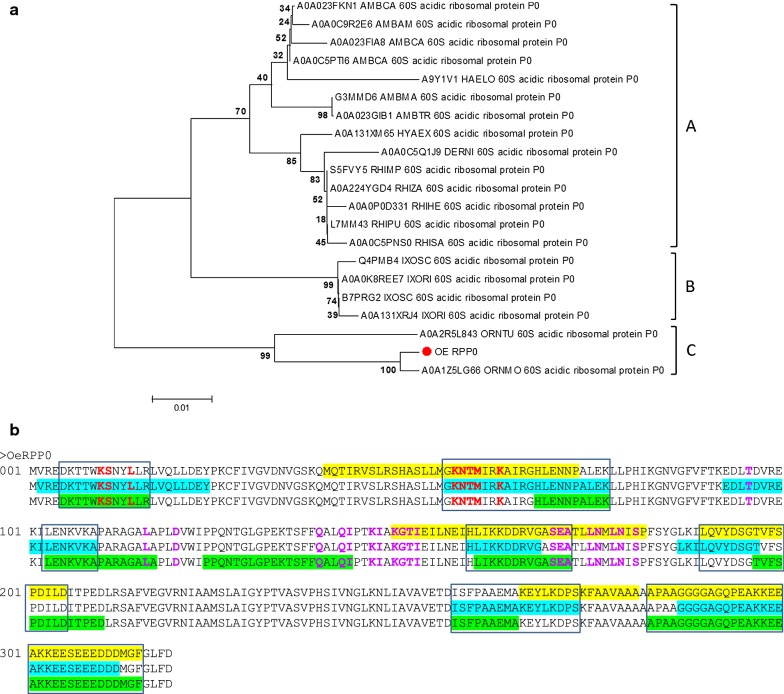
**a** Neighbour-joining analysis of the phylogenetic relationship of the Acidic 60S Ribosomal protein P0 orthologues in ticks. OeRPP0, *Ornithodoros erraticus* ribosomal protein P0 (red dot). Uniprot entry names are shown for the other sequences: AMBAM, *Amblyomma americanum*; AMBCA, *A. cajennense*; AMBMA, *A. maculatum;* AMBTR; *A. triste*; DERNI, *Dermacentor nitens*; HAELO, *Haemaphysalis longicornis*; HYAEX, *Hyalomma excavatum*; IXORI, *Ixodes ricinus*; IXOSC, *I. scapularis*; ORNER, *Ornithodoros erraticus*; ORNMO, *O. moubata*; ORNTU, *O. turicata*; RHIHA, *Rhipicephalus haemaphysaloides*; RHIMP, *R. microplus*; RHIPU, *R. pulchellus*; RHISA, *R. sanguineus*; RHIZA, *R. zambeziensis*. Evolutionary distances were computed using the Poisson correction method. Branch support values (10,000 bootstraps) for the nodes are indicated. **b** Linear B-cell epitope predictions for OeRPP0. The sequence of the protein is represented in triplicate showing the ABCpred (yellow), BCEpred (blue) and BepiPred-2.0 (green) predictions. The amino acids in epitopes predicted by at least two algorithms were highlighted in boxes. The 8 amino acids that form the 23S rRNA interface are in bold red and the 20 ones that form the interface with the L7/L12 ribosomal proteins are in bold purple

The phylogenetic analysis confirmed the close relationship among all of these RPP0s and grouped them into three main clusters, which respectively include the RPP0 from Metastriata (A), Prostriata (B) and Argasidae (C), supported by 70%, 99% and 99% bootstrap values (Fig. 3a).

Each lineal B-cell epitope prediction algorithm identified a set of different but overlapping linear B-cell epitopes throughout the whole protein sequence. Up to 7 epitopes were predicted by two or the three algorithms, and thus considered consensus predicted linear B-cell epitopes. The longest epitope spanned 26 amino acid residues close to the carboxy-end, inside a highly unstructured region of the protein (Fig. 3b).

The 3D modelling of OeRPP0 modelled up to 269 residues (84%) of its amino acid sequence with 100% confidence by the single highest scoring 3D template. Additional file 4: Figure S3b presents the 3D model of OeRPP0, showing the highly conserved secondary structure of RPP0s and the predicted linear B-cell epitopes localised on the protein surface, where they cover the whole putative interface with the 23S rRNA and most part of the interface with the L7/L12 ribosomal proteins. The longest predicted epitope could not be included in the 3D model because it mapped outside the modelled region. For this protein, the whole amino acid sequence was cloned and expressed as recombinant candidate antigen.

### Secreted peptides and proteins: selected candidate OePK4

Forty-six transcripts annotated as “secreted peptide” or “secreted protein” were mined from the *O. erraticus* midgut transcriptome, very few of them having functional annotation (Additional file 1: Table S1). Twenty-one were significantly upregulated (fold-change > 2) after feeding and 11 of them encoded antigenic proteins (Vaxijen score > 0.5). Among the predicted antigens, transcript ci|000079280 showed simultaneously the highest fold-change upon feeding (3954) and the highest Vaxijen score (1.0632). Accordingly, it was selected as candidate protective antigen. This transcript encodes a 109 amino acid long polypeptide without functional annotation (Uniprot: A0A293MVU8).

Searching Uniprot and NCBInr databases for tick orthologues of A0A293MVU8 provided numerous related sequences, from which the top 10 matches were selected; they all showed E-values < 10^−7^ and sequence identities between 32% and 58%, and included 4 argasid and 6 ixodid sequences. Multiple alignment of A0A293MVU8 and these proteins showed that A0A293MVU8 has a poorly conserved amino-terminal region (residues 1–57) and a more conserved carboxy-terminal region (residues 58–109), reaching in this region more than 50% sequence identity to the Q4PMD7, B7PVH8 and B7PUK6 proteins of *Ixodes scapularis* (Additional file 5: Figure S4).

**Fig. 4:**
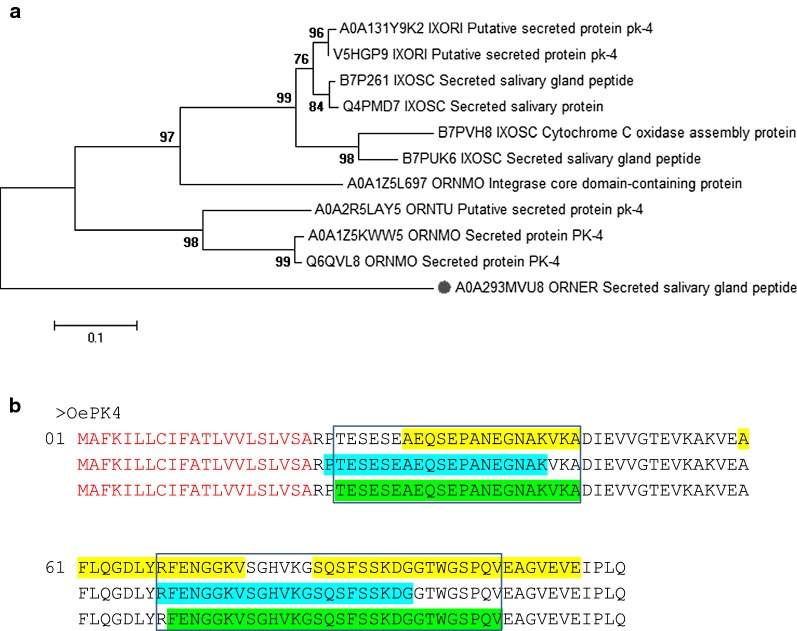
**a** Neighbour-joining analysis of the phylogenetic relationship of A0A293MVU8_ORNER (OePK4, red dot) and orthologous proteins. Uniprot entry names are shown: IXORI, *Ixodes ricinus*; IXOSC, *I. scapularis*; ORNER, *Ornithodoros erraticus*; ORNMO, *O. moubata*; ORNTU, *O. turicata*. Evolutionary distances were computed using the Poisson correction method. Branch support values (10,000 bootstraps) for the nodes are indicated. **b** Linear B-cell epitope predictions for OePK4. The sequence of the protein is represented in triplicate showing the ABCpred (yellow), BCEpred (blue) and BepiPred-2.0 (green) predictions. The amino acids in epitopes predicted by at least two algorithms are highlighted in boxes. The signal peptide is depicted in red

Phylogenetic analysis of these proteins grouped them in two main clusters, supported by 97% and 98% bootstrap values, but placed A0A293MVU8 outside these clusters (Fig. 4a). None of these proteins were found in the Pfam, Prosite and InterPro databases of protein families, domains and functional sites (data not shown). However, all of them but B7P261 and Q4PMD7 belong to the Uniprot “Uniref_cluster: Cytochrome *c* oxidase assembly protein”, whose representative member is B7PVH8. Sequence identity between A0A293MVU8 and B7PVH8 is 57.8%, which might suggest a functional relation of A0A293MVU8 with the mitochondrial respiratory chain and redox-linked proton pumping. Despite this, since most of the A0A293MVU8 orthologues found were annotated as “secreted protein PK4”, we termed A0A293MVU8 as OePK4.

Topology predictions for OePK4 predicted a secreted polypeptide with a signal peptide of 21 amino acids long, without transmembrane domains or GPI anchors. 3D modelling of OePK4 only modelled 16 amino acids (15%) with 29.1% confidence, thus it was no longer considered (not shown).

Linear B-cell epitope prediction tools forecasted two epitopes for OePK4. The first epitope was located immediately downstream the signal peptide and the second in the carboxy-terminal half of the protein, where OePK4 shows the higher sequence identity to their orthologues in other tick species (Fig. 4b; Additional file 5: Figure S4). Thus, a truncated version of OePK4 without signal peptide was cloned and expressed as recombinant candidate antigen.

### Recombinant protein production

The five candidates selected were all subcloned and expressed into the pQE-30 vector. However, the expression of CHI and TSP1 failed and they were subcloned and expressed on the pGEX-4T1 vector.

Finally, the CHI-GST and TSP1-GST fusion proteins, the full-length recombinant RPP0, the truncated version (without signal peptide) of PK4 and the long extracellular loop of TSP2 were all successfully expressed and purified (Additional file 6: Figure S5). All of them migrated in SDS-PAGE gels as single bands of the predicted molecular weight (MW), except PK4 and TSP2, which showed experimental MWs (13.5 and 16.5 kDa, respectively) somewhat larger than their predicted MWs (11.5 and 11.1 kDa, respectively). Hence, the identity of these recombinants was confirmed by LC-MS/MS mass spectrometry analysis of the corresponding gel band (not shown).

### Vaccine trial 1: humoral immune response to recombinant antigens and protective effects against tick infestations

**Fig. 5:**
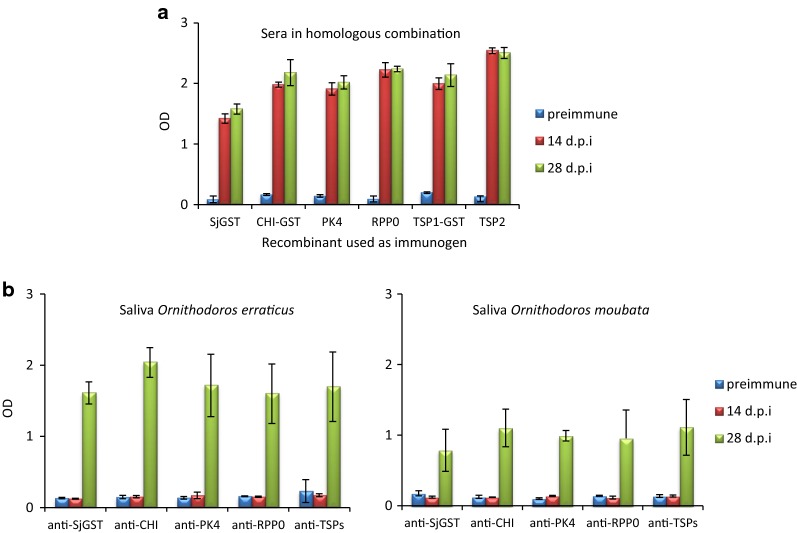
ELISA. IgG antibody response in rabbits vaccinated with recombinant antigens SjGST (control), OeCHI, OePK4, OeRPP0 and OeTSP1 + OeTSP2 (TSPs). **a** Reactivity of rabbit sera to the homologous recombinant antigen. **b** Reactivity of rabbit sera to the saliva of *Ornithodoros erraticus* and *Ornithodoros moubata.* Values are the average OD ± SD at 492 nm from each rabbit group. Sera were taken before immunisation (preimmune), 14 days post-immunisation, immediately before the infestation with ticks (14 dpi) and 14 days post-infestation (28 dpi), and were used at 1/300 dilution

The rabbits vaccinated with the five recombinant antigens developed strong antibody responses to the homologous antigen. The sera obtained 14 dpi, immediately before the infestation, showed antibody titres higher than 1/12,800 and optical densities (OD) higher than 2.0. Control rabbits immunised with SjGST reacted to this recombinant with antibody titres close to 1/6400 and OD around 1.5 (Fig. 5a).

The sera obtained 28 dpi (14 days post-infestation) reacted with almost identical intensity to the recombinant antigens than the sera obtained 14 dpi did, suggesting lack of cross-reactivity between the recombinant antigens and the salivary proteins inoculated during feeding (Fig. 5a). Conversely, no sera obtained 14 dpi before the infestation reacted to the saliva of *O. erraticus* or *O. moubata* (Fig. 5b).

**Fig. 6:**
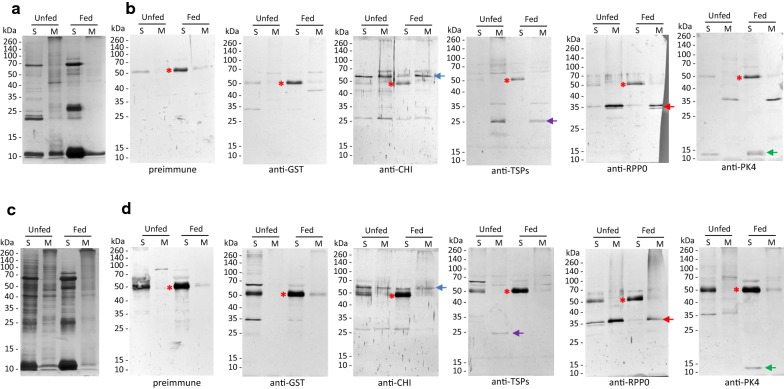
**a**, **c** Coomassie Blue-stained 15% SDS-PAGE gels showing the soluble (S) and membrane (M) proteins of the midgut from *Ornithodoros erraticus* (**a**) and *O. moubata* (**c**) female ticks taken before feeding (Unfed) and 48 h after engorgement (Fed). **b, d** Western blots: antigens revealed by the sera from the rabbits vaccinated with the recombinant antigens SjGST, OeCHI, OeTSP1 + OeTPS2 (TSPs), OeRPP0 and OePK4 on the protein extracts from *O. erraticus* (**b**) and *O. moubata* (**d**). Sera were taken before immunisation (preimmune) and 14 days post-immunisation, immediately before the infestation with ticks. Red asterisks: IgG heavy chain from the rabbit host ingested with blood. Arrows: native forms of OeCHI (55 kDa, blue), OeTSPs (25.9–26.4 kDa, purple) OeRPP0 (34.7, red) and OePK4 (11.5, green) recognised by the immune sera

The reactivity of the rabbit IgG antibody response to native forms of the OeCHI, OeTSP1, OeTSP2, OeRPP0 and OePK4 proteins in the midgut extracts from *O. erraticus* and *O. moubata* was analysed in ELISA (Additional file 7: Figure S6) and western blot (Fig. 6). According to the ELISA results, the immune sera showed low reactivity to the midgut extracts of *O. erraticus*, and even lower reactivity to the midgut extracts of *O. moubata* (Additional file 7: Figure S6). Part of this reactivity was due to the nonspecific recognition of host IgG, which was also recognised by the preimmune sera and the sera anti-SjGST (Fig. 6). Overall, the immune sera showed higher reactivity to membrane proteins than to soluble proteins from both fed and unfed *O. erraticus* and *O. moubata* females, except for the anti-PK4 sera which showed the opposite behaviour, reacting more intensely to the soluble proteins (Fig. 6, Additional file 7: Figure S6).

On western blot, the anti-CHI serum pool reacted with a 55 kDa band compatible with the native OeCHI protein in every protein extract analysed, namely, extracts of soluble and membrane proteins from fed and unfed *O. erraticus* (Fig. 6b) and *O. moubata* females (Fig. 6d). This indicates that OeCHI and its orthologue in *O. moubata* share cross-reacting epitopes and a similar localisation in the enterocyte, including in the plasma membrane and extracellular region or secreted.

The anti-TSPs serum pool reacted with a band of 26 kDa, compatible with both the OeTSP1 and OeTSP2 native proteins, on the membrane proteins from fed and unfed *O. erraticus* and the membrane proteins from unfed *O. moubata* females. This is in agreement with the membrane predicted localisation of TSPs and also indicates cross-reactive epitopes between orthologous TSP proteins in these two *Ornithodoros* species.

The anti-RPP0 sera recognised a 34.7 kDa intense band, compatible with the OeRPP0 native protein, on the extracts of membrane proteins of both *Ornithodoros* species and physiological stages (fed, unfed), and a similar but fainter band on the parallel extracts of soluble proteins (Fig. 6b, d). Thus, OeRPP0 and its native orthologue in *O. moubata* share cross-reacting epitopes and localisation in the enterocyte including cytosolic large ribosomal subunit, endoplasmic reticulum and extracellular region or secreted.

Finally, the anti-PK4 serum pool recognised one band of 11.5 kDa compatible with the predicted native OePK4 in the extracts of soluble proteins from fed and unfed *O. erraticus* (Fig. 6b) and soluble proteins from fed *O. moubata* (Fig. 6d), showing that OePK4 shares cross-reactive epitopes with orthologous proteins in *O. moubata*.

No significant differences were observed between the two control groups (adjuvant, SjGST) for any species and developmental stage in any of the parameters evaluated (Tables 2, 3). Accordingly, these two groups were combined and treated as a single control group.

**Table 2:** Effect of the vaccination with recombinant proteins CHI-GST, TSP1-GST + TSP2, RPP0, and PK4 administered in Montanide ISA 50 V2 on *O. erraticus* specimens fed on control and vaccinated rabbits

Parameter	Developmental stage	Control Merged	Control Montanide	Control SjGST	CHI-GST (% reduction)	TSP1-GST + TSP2 (% reduction)	RPP0 (% reduction)	PK4 (% reduction)
Ingested blood (mg)	Males	3.7 ± 0.5	3.7 ± 0.1	3.7 ± 0.7	3.6 ± 0.1 (2.7)	3.8 ± 0.2	4.1 ± 0.2	3.4 ± 0.3 (8.1)
	Females	13.0 ± 1.1	13.0 ± 3.1	13.0 ± 1.2	13.3 ± 0.6	13.3 ± 1.2	12.8 ± 2.0 (1.5)	12.5 ± 0.2 (3.8)
	Nymphs-3	3.3 ± 0.5	3.2 ± 0.4	3.5 ± 0.5	3.0 ± 0.5 (9.1)	3.9 ± 0.4	3.3 ± 0.5	3.1 ± 0.7 (6.1)
Mortality (%)	Males	0.6 ± 1.4	0 ± 0	1.1 ± 1.8	0 ± 0	0 ± 0	0 ± 0	0 ± 0
	Females	0 ± 0	0 ± 0	0 ± 0	0 ± 0	0 ± 0	2.1 ± 3.0 (2.1)	2.1 ± 3.0 (2.1)
	Nymphs-3	4.3 ± 2.9	6.0 ± 2.0	2.7 ± 2.7	1.3 ± 1.0	10.0 ± 12.9 (5.7)	1.4 ± 1.0	4.1 ± 3.5
Moulting (%)	Nymphs-3	60.5 ± 8.9	61.1 ± 9.8	59.8 ± 9.0	42.3 ± 3.0 (30.1)**	45.5 ± 15.2 (24.8)**	38.1 ± 10.7 (37.1)**	31.8 ± 19.8 (47.4)**
Oviposition (no. eggs/female)	Females	66.4 ± 6.0	67.2 ± 8.5	65.6 ± 3.5	61.6 ± 3.4 (7.2)	50.0 ± 4.5 (24.7)**	48.0 ± 6.1 (27.7)**	54.2 ± 6.0 (18.4)**
Fertility (no. nymphs/female)	Females	55.5 ± 5.7	56.8 ± 6.3	54.3 ± 5.3	53.7 ± 1.6 (3.2)	32.5 ± 4.4 (41.4)**	36.6 ± 2.7 (34.1)**	41.5 ± 6.7 (25.2)**
Efficacy (%)					30.2	56.0	57.5	57.8

*Notes*: Results are shown as mean ± standard deviation for each rabbit group. Means were compared between ticks fed on vaccinated and control rabbits (merged) by one-way ANOVA followed by the Dunnett’s t-test. In parentheses, % of change in the corresponding parameter respect to the control: percentage of reduction for ingested blood, moulting, oviposition and fertility; increase for mortality

** *P* < 0.01

**Table 3:** Effect of the vaccination with recombinant proteins CHI-GST, TSP1-GST + TSP2, RPP0, and PK4 administered in Montanide ISA 50 V2 on *O. moubata* specimens fed on control and vaccinated rabbits

Parameter	Developmental stage	Control Merged	Control Montanide	Control SjGST	CHI-GST (% reduction)	TSP1-GST + TSP2 (% reduction)	RPP0 (% reduction)	PK4 (% reduction)
Ingested blood (mg)	Males	32.2 ± 6.0	32.1 ± 5.1	32.3 ± 6.5	29.4 ± 0.4 (8.4)	30.9 ± 1.1 (3.8)	30.1 ± 2.5 (6.2)	26.4 ± 1.0 (17.8)*
	Females	203.6 ± 20.4	204.9 ± 20.7	202.3 ± 20.1	159.0 ± 133.0 (22.4)*	198.1 ± 14.6 (3.3)	193.6 ± 18.0 (5.5)	192.2 ± 16.0 (6.2)
	Nymphs-3	45.8 ± 6.1	45.8 ± 4.5	45.9 ± 7.0	41.5 ± 2.9 (9.3)	44.1 ± 1.2 (3.6)	45.6 ± 5.7 (0.5)	37.3 ± 1.2 (18.6)*
Mortality (%)	Males	6.4 ± 4.5	5.9 ± 7.1	6.8 ± 0.3	6.7 ± 7.3	6.7 ± 2.8	6.0 ± 8.5	4.4 ± 1.5
	Females	5.5 ± 2.7	6.6 ± 0	4.3 ± 3.3	6.3 ± 0	4.2 ± 3.0	2.1 ± 3.0	2.1 ± 3.0
	Nymphs-3	2.0 ± 1.7	2.0 ± 2.0	2.0 ± 1.7	0.7 ± 1.0	3.3 ± 3.4 (1.3)	0.7 ± 0.9	1.9 ± 1.6
Moulting (%)	Nymphs-3	67.2 ± 1.7	66.2 ± 1.7	68.2 ± 1.3	63.4 ± 36.5 (4.1)	61.8 ± 8.4 (6.6)	72.0 ± 4.0	57.4 ± 3.6 (13.3)*
Oviposition (no. eggs/female)	Females	197.3 ± 18.8	203.0 ± 23.0	191.0 ± 12.0	158.0 ± 2324 (22.2)**	182.0 ± 14.0 (10.3)	192.0 ± 14.0 (5.4)	191.0 ± 6.7 (5.9)
Fertility (no. nymphs/female)	Females	179.9 ± 15.8	181.0 ± 21.1	179.0 ± 20.0	148.0 ± 21.0 (18.2)*	170.0 ± 15.0 (6.0)	179.0 ± 20.0 (1.1)	180.0 ± 7.0 (0.5)
Efficacy (%)					19.6	11.1	0	8.1

*Notes*: Results are shown as mean ± standard deviation for each rabbit group. Means were compared between ticks fed on vaccinated and control rabbits (treated with adjuvant alone) by one-way ANOVA followed by the Dunnett’s t-test. In parentheses, % of change in the corresponding parameter respect to the control: percentage of reduction for ingested blood, moulting, oviposition and fertility; increase for mortality

* *P* < 0.05, ** *P* < 0.01

On the *O. erraticus* ticks, the anti-CHI response caused a significant reduction on nymph moulting, while the anti-TSPs, anti-RPP0 and anti-PK4 responses induced significant decreases in nymph moulting, female oviposition and female fertility. Accordingly, the vaccine efficacy of the OeCHI, OeTSPs, OeRPP0 and OePK4 recombinant antigens against *O. erraticus* infestations was 30.2, 56.0, 57.5 and 57.8%, respectively (Table 2).

On the *O. moubata* ticks, the recombinant antigens induced low protective effects, being mostly non-significant (Table 3). Only the anti-CHI response caused significant reductions in female feeding and reproduction; the anti-PK4 response caused significant reductions in nymph feeding and moulting, while the anti-TSPs response caused generalised non-significant reductions in tick feeding, nymph moulting and female reproduction. This resulted in lower global vaccine efficacies for the OeCHI, OeTSPs, OeRPP0 and OePK4 recombinant antigens against *O. moubata* infestations: 19.6, 11.1, 0 and 8.1%, respectively.

### Vaccine trial 2: humoral immune response and protective effects induced by jointly administered candidate antigens against soft tick infestations

The immune sera from all vaccinated rabbits showed IgG antibody titres higher than 1/12,800 to each single recombinant antigen and around 1/3200 to the OeSEL synthetic peptide, confirming that all animals developed strong humoral responses.

Table 4 summarises the protective effects induced by the multicomponent vaccine against tick infestations. The anti-*O. erraticus* protective response was more intense than that of trial 1, and mainly impacted female reproduction and nymph mortality, resulting in an increased global vaccine efficacy (66.3%), which was 15% higher than the best protection reached with candidates tested individually. Regarding *O. moubata,* the vaccine cocktail induced similar but weaker effects than those in *O. erraticus*, namely, significant reductions in female reproduction and non-significant reductions in feeding performance, which resulted in a 25.6% vaccine efficacy, which was 30% higher that the best protection reached with individual candidates.

**Table 4:** Effect of the vaccination with a multiantigenic formulation containing candidate antigens OeTSP1, OeTSP2, OeRPP0, OePK4 and OeSEL in Montanide ISA 50 V2 on *O. erraticus* and *O. moubata* ticks fed on control and vaccinated rabbits

Parameter	Developmental stage	*Ornithodoros erraticus*		*Ornithodoros moubata*	
		Control	Vaccinated (% reduction)	Control	Vaccinated (% reduction)
Ingested blood (mg)	Males	3.1 ± 0.3	3.2 ± 0.1	26.2 ± 2.2	26.9 ± 2.1
	Females	9.2 ± 0.8	9.3 ± 1.1	191.4 ± 57.1	180.1 ± 12.9 (5.9)
	Nymphs-3	2.1 ± 0.2	2.0 ± 0.2 (4.8)	5.5 ± 0.7	5.3 ± 0.6 (3.6)
Mortality (%)	Males	2.8 ± 5.3	2.2 ± 3.2	6.1 ± 2.6	14.1 ± 3.2 (8.0)
	Females	1.1 ± 2.7	0 ± 0	9.0 ± 5.6	8.9 ± 8.4
	Nymphs-3	0.4 ± 1.0	13.2 ± 7 (12.8)**	2.8 ± 2.2	3.3 ± 1.0 (0.5)
Moulting (%)	Nymphs-3	62.3 ± 10.7	61.8 ± 9.0	96.8 ± 1.9	94.4 ± 4.8
Oviposition (no. eggs/female)	Females	62.2 ± 7.3	26.6 ± 3.3 (57.0)**	200.0 ± 21.0	146.0 ± 12.9 (27.0)**
Fertility (no. nymphs/female)	Females	54.1 ± 5.8	21.5 ± 2.7 (60.2)**	182.0 ± 23.0	137.5 ± 11.0 (24.5)**
Efficacy (%)			66.3		25.6

*Notes*: Results are shown as the mean ± standard deviation for each rabbit group. Means were compared between ticks fed on vaccinated and control rabbits by one-way ANOVA followed by the Dunnett’s t-test. In parentheses, % of change in the corresponding parameter respect to the control: percentage of reduction for ingested blood, moulting, oviposition and fertility

** *P* < 0.01

## Discussion

Identifying highly protective antigens for tick vaccines development may be tackled by selecting protein candidates that play essential biological functions and share conserved sequence motifs to allow the simultaneous control of different tick species [1, 68].

In the present study, we focused on midgut chitinases, tetraspanins and RPP0 because these proteins are involved in important midgut physiological processes; several publications highlight their potential as vaccine candidates to control parasite infections [28, 29, 33, 42]. Additionally, and despite their unknown functions, we also focused on secreted proteins/peptides because of their accessibility to host antibodies ingested in blood, which make them the first-election candidate antigens for tick vaccine design [69, 70].

Topological analysis of the five selected candidates (chitinase, tetraspanins, RPP0 and secreted protein/peptide) confirmed the transmembrane location and extracellular region/loops of OeCHI and OeTSPs (Additional file 2: Figure S1b and Additional file 3: Figure S2d, respectively), as well as the cytoplasmic location of OeRPP0, and the secreted nature of OePK4. Lineal B-cell epitope predictions for the five candidates also verified the presence of this kind of epitope on the extracellular region/loops of OeCHI and OeTSPs (Figs. 1b, 2b) and throughout the amino acid sequence of OeRPP0 (Fig. 3b) and OePK4 (Fig. 4b) supporting their antigenicity. Interestingly, the 3D modelling of the candidates (except OePK4) showed that the predicted B-cell epitopes localize on the protein surface (Additional file 2: Figure S1c, Additional file 3: Figure S2e and Additional file 4: Figure S3b, respectively) where they could be easily reached by host antibodies. The multiple alignment of each candidate with its orthologues in other argasid and ixodid tick species showed that they share conserved structural and sequence motifs, including most of their antigenic extracellular regions (Additional file 2: Figure S1a, Additional file 3: Figure S2a, Additional file 4: Figure S3a and Additional file 5: Figure S4, respectively. This could facilitate the simultaneous targeting of different tick species if the candidates induce cross-protective immune responses.

The five candidates triggered robust immune responses in rabbits demonstrating high immunogenicity (Fig. 5a), which is in agreement with the linear B-cell epitope predictions and VaxiJen prediction of antigenicity for all of them except OeRPP0. These responses specifically recognised the inducing recombinant protein but did not cross-react with saliva of *O. erraticus* nor *O. moubata* (Fig. 5b). Consequently, the tick bites did not boost the vaccine-induced humoral responses (Fig. 5a), showing that natural tick-host contacts would not serve as boosting antigen doses in immunised hosts.

The immune sera to OeCHI and to OeTSPs recognised their respective native protein targets on the membrane protein extracts of *O. erraticus* ticks (Fig. 6b), confirming the presence of these proteins in the midgut proteome [23] and their predicted location to enterocyte membranes. The sequence identity shown by OeCHI and OeTSP1 to homologous proteins from *O. moubata* (Additional file 2: Figure S1a and Additional file 3: Figure S2a, respectively) can explain the fact that anti-OeCHI and anti-OeTSPs sera also recognised native chitinases and tetraspanins on the midgut protein extracts of *O. moubata* (Additional file 7: Figure S6), anticipating the possibility of some cross-species protection.

The anti-OeCHI immune response provided significant protection (30.1%) to *O. erraticus* by reducing the moulting rate of nymphs without affecting other parameters (Table 2). This result is similar to the protective effect reported by [28] in mice vaccinated with a recombinant form of the *H. longicornis* chitinase CHT1, and strongly suggests that OeCHI is involved in moulting. Notably, the anti-OeCHI immune response also provided significant cross-protection to *O. moubata* (19.6%), but it consisted of a general reduction in tick feeding performance, significant only in females, and significant reductions in female oviposition and fertility, without affecting nymph moulting (Table 3). These results resemble the phenotypic effect observed after RNAi gene knockdown of a salivary chitinase of *A. americanum* supposedly involved in tick feeding and cement cone stability maintenance [29]. Accordingly, it appears that the anti-OeCHI immune response recognises cross-reactive epitopes in an *O. moubata* chitinase that is likely involved in tick feeding and functionally distinct of the OeCHI identified in the present study. Based on the findings and data mining of this study, it seems that the tick GH-18 family has multiple members with different functions, and suggests that OeCHI might be a useful vaccine candidate antigen for the control of ornithodoros ticks.

Regarding tetraspanins, they are scaffold proteins that may participate in numerous and important intra- and inter-cellular biological processes due to their ability to interact with other proteins [32, 71]. The anti-OeTSPs immune response provided significant protection (56%) to *O. erraticus* and a notably lower cross-protection (11.1%) to *O. moubata*, which is in agreement with the lower reactivity of the anti-OeTSPs sera to the *O. moubata* midgut extracts (Fig. 6, Additional file 7: Figure S6). The protective effect was, however, qualitatively similar in both species: basically, reductions in nymph moulting and female oviposition and fertility (Tables 2, 3). These results suggest the involvement of OeTSP1 and/or OeTPS2 in these processes, although further investigation is needed to determine the particular functions of these two tetraspanins. OeTSP1 and OeTSP2 only share 27.3% sequence identity to each other (Additional file 3: Figure S2c) and belong to different phylogenetic clades (Fig. 2a), suggesting that they may play different functions. Additionally, OeTSP1 is more conserved among tick tetraspanins than OeTSP2 (85–94.7% and 30–58%, respectively; Additional file 3: Figure S2a, b), suggesting that OeTSP1 could be the main cross-reactive antigen, and hence more useful for the simultaneous control of different tick species. Nevertheless, the present results show that these two tetraspanins, OeTSP1 and OeTPS2, can be suitable candidate antigens for vaccines aimed at the control of ornithodoros ticks, and establish the tick tetraspanin family as a source of potential vaccine targets for the first time.

The anti-OeRPP0 antibodies recognised the native form of the protein on the midgut membrane extracts of *O. erraticus* (Additional file 7: Figure S6) confirming its presence in the proteome [23] and its location as part of the ribosomes and the endoplasmic reticulum in the cytoplasm. As expected because of its high sequence identity (99.4%, Additional file 4: Figure S3a), the anti-OeRPP0 also recognised the *O. moubata* RPP0 orthologue on the membrane extracts of this species (Fig. 6d).

The anti-OeRPP0 response induced significant protection (57.5%) to *O. erraticus* by reducing nymph moulting and female oviposition and fertility without any protective effect to *O. moubata,* despite the strong recognition of its RPP0 orthologue. This higher sensitivity of *O. erraticus vs O. moubata* to vaccines based on midgut antigens has already been observed and discussed by our team in previous studies, where it was reasoned that there may be particular factors in *O. moubata* (anatomical, physiological, molecular or microbial) that decrease the accessibility of immune effectors to their targets thus reducing the vaccine efficacy of the midgut concealed antigens against this species [20–22]. The protection provided by OeRPP0 to *O. erraticus* was lower than the 90–96% reached with peptide pP0 to ixodid ticks by [42, 43], which consisted in reduced feeding performance, reduced moulting and reproduction, and increased mortality. RPP0 is primarily involved in protein synthesis in ribosomes, so impairing this function might impact all the tick physiological processes dependent on the correct functioning of ribosomes. These would include the synthesis of salivary proteins and new cuticle that occur during feeding in ixodid but not argasid ticks [72, 73] and the increase in protein expression that take place during moulting and reproduction in both tick families. The higher number of physiological processes affected in ixodids would explain, at least partially, the higher impact in ixodids *vs* argasids of the RPP0-based vaccines. However, RPP0 is a multifunctional protein, with a complex and incompletely understood biology [44, 45], which may complicate the explanation of how the host anti-RPP0 antibodies exercise their protective effects. Although further studies are needed to disclose which other functions of tick RPP0 are blocked by host antibodies and how the antibodies reach the target protein, the present results reinforce the notion that tick RPP0 is a good broad spectrum candidate for tick vaccines, including argasids.

Regarding the selected “secreted peptide A0A293MVU8 (OePK4)”, it grouped together with several PK-4 secreted proteins and secreted salivary gland peptides in the phylogenetic analysis. All of these proteins/peptides lacked functional annotations, except B7PVH8, which is annotated as “Cytochrome *c* oxidase assembly protein”, suggesting a functional association between OePK4 and the mitochondrial respiratory chain (Fig. 4a). However, this association does not seem very probable since, far from being an integral component of mitochondrial inner membrane, OePK4 is a soluble secreted protein, as indicated by its *in silico* analysis and confirmed by the fact that it is recognised by the anti-OePK4 sera on the soluble fraction of midgut proteins in *O. erraticus* and *O. moubata* (Fig. 6b, c). Additionally, according to its description in the mialome (Additional file 1: Table S1), OePK4 would have a salivary origin and it would have reached the midgut with ingested saliva, where it would play hitherto unknown functions. This origin may however be controversial because the anti-OePK4 antibodies did not reacted to saliva proteins and natural contacts with ticks did not increase the reactivity of anti-OePK4 sera (Fig. 5).

The anti-OePK4 response provided 57.8% significant protection to *O. erraticus* by reducing nymph moulting and female oviposition and fertility, and 8.1% cross-protection to *O. moubata* by reducing tick feeding, tick survival and nymph moulting. These results highlight the potential of OePK4 as a protective candidate antigen and open the door to further studies to determine its function(s), tissue expression and expression regulation, and the mechanisms underlying its protective effects.

Finally, the results of trial 2 demonstrate that a combination of recombinant multi-epitope antigens targeting different tick physiological mechanisms increased vaccine efficacy compared with individual antigens. This highlights the potential usefulness and convenience of developing multicomponent vaccines for the control of ticks.

## Conclusions

The recently obtained transcriptomic and proteomic data from the *O. erraticus* midgut has allowed us to apply a function-based approach to select candidate protective protein antigens from the tick midgut: one chitinase, two tetraspanins, the ribosomal protein P0 and one secreted protein PK4. The vaccination of rabbits with these candidates confirmed their predicted immunogenicity, since they all induced strong humoral immune responses. All candidates showed medium level protection against *O. erraticus* ticks, and all but RPP0 showed partial cross-protection against *O. moubata*. Protective effects were assumed to be the result of an antibody-mediated loss of function of the antigen targets. The results of the present study support that at least one chitinase and the ribosomal protein P0 from *Ornithodoros* ticks are promising protective antigens that might be included in vaccines aimed at control of multiple tick species. They also provide new protective antigens from argasids, namely, tetraspanins OeTSP1 and OeTSP2, and secreted protein PK4, that belong to protein families never tested before as protective antigens in ticks, which deserve further investigation. Finally, these results demonstrate that multicomponent vaccines increased vaccine efficacy compared with the individual antigens. New protective antigens from *Ornithodoros* spp. are still needed and will probably be identified by targeting tick proteins playing relevant biological functions for tick survival and pathogen-tick-host- interactions. Novel strategies for integrating multi-omics tools and data would facilitate a greater understanding of parasitic diseases. Proteogenomics approaches aimed at pathogen-tick-host-tick interactions will certainly allow abundant omics data to be acquired, the integration and analysis of which with modern functional studies will facilitate the identification of interesting targets and their valuation as vaccine candidate antigens.

## Supplementary information

**Additional file 1: Table S1.** Transcriptomic data for chitinases, tetraspanins, ribosomal protein P0 and secreted proteins/peptides from the *Ornithodoros erraticus* midgut.

**Additional file 2: Figure S1.** Tick chitinases sequence alignment and OeCHI topology prediction.

**Additional file 3: Figure S2.** Tick tetraspanins sequence alignment and OeTSPs topology prediction.

**Additional file 4: Figure S3.** Tick RPP0 sequence alignment and OeRPP0 topology prediction.

**Additional file 5: Figure S4.** Alignment of the amino acid sequences of A0A293MVU8_ORNER (OePK4) and orthologous peptides and proteins.

**Additional file 6: Figure S5.** Obtaining of candidate antigens as recombinant proteins.

**Additional file 7: Figure S6.** Analysis of reactivity of the vaccinated rabbit sera by ELISA.

## Data Availability

The data supporting the conclusions of this article are provided within the article and its additional files. Raw data are available from the corresponding author upon reasonable request. The midgut transcriptome data used during this study were deposited in DDBJ/ENA/GenBank under accession number GFWV00000000 as a Transcriptome Shotgun Assembly project (BioProject: PRJNA401392).

## Abbreviations

ABC: ATP-binding cassette transporter
ANOVA: analysis of variance
ASF: African swine fever
CHI: chitinase
FPKM: fragments per kilobase of transcript per million mapped reads
GH18: glycoside hydrolase-18
GPI: glycosylphosphatidylinositol
GST: glutathione S-transferase
LC-MS/MS: liquid chromatography-tandem mass spectrometry
NCBInr: National Center for Biotechnology Information non redundant
PBS: phosphate-buffered saline
PK4: secreted protein PK-4
RNAi: RNA interference
RPP0: 60S acidic ribosomal protein P0
RT-PCR: reverse transcription-polymerase chain reaction
SDS-PAGE: sodium dodecyl sulphate polyacrylamide gel electrophoresis
SEL: selenoprotein
SjGST: *Schistosoma bovis* glutathione S-transferase
TBRF: tick-borne relapsing fever
TPBS: PBS supplemented with 0.05% Tween 20
TSP: tetraspanin
