# Supplementary material for: In silico selection of functionally important proteins from the mialome of Ornithodoros erraticus ticks and assessment of their protective efficacy as vaccine targets
Source: Parasit Vectors. 2019 Oct 30;12:508. doi: 10.1186/s13071-019-3768-1 (PMC6822432; doi:10.1186/s13071-019-3768-1)

Additional file 2: Figure S1. a Alignment of the amino acid sequences of tick chitinases belonging to the glycoside hydrolase-18 (GH-18) family. OeCHI, *Ornithodoros erraticus* chitinase. Uniprot entry names are shown for the other sequences: AMBAM, *Amblyomma americanum*; IXORI, *Ixodes ricinus*; IXOSC, *I. scapularis*; ORNER, *Ornithodoros erraticus*; ORNMO, *O. moubata*; ORNTU, *O. turicata*; RHIPU, *Rhipicephalus pulchellus*; RHISA, *R. sanguineus*; RHIZA, *R. zambeziensis*. The amino acids conserved in all sequences are labelled with asterisks (*); the conservative and semi-conservative substitutions are labelled with colons (:) and periods (.), respectively. The four signature amino acid sequence motifs of the arthropod GH-18 chitinases are highlighted in yellow. The GH18 family active site is underlined. On the OeCHI sequence, the signal peptide and the carboxy-terminal transmembrane domain are highlighted in blue, and the alanine 35 and asparagine 398, both in purple, are the start and end points of the GH18 catalytic domain. The percentage of sequence identity showed by OeCHI to the other tick GH-18 family members is indicated in the column on the lower right corner. **b** Topology predictions for OeCHI using the TMHMM server. The values indicate the amino acids forming part of each region. **c** Three-dimensional modelling. Left: 3D model of the GH-18 catalytic domain of the OeCHI showing the (α/β)8-TIM-barrel as a core structure. Right: 3D model showing the linear B-cell epitope predictions on the molecule surface (yellow).

a

```
OeCHI               ------------------------MLGSEAVRL--LLLSLCVVSAV--------------    20
B7P5Q8_IXOSC        -------------------------------MM------------------LSAPVETTT    11
B7QIK8_IXOSC        -------------------------------MV------------------GVAPS----    7
A0A1Z5L6Y6_ORNMO    ---------------------------MKRLVVLLL--STLYAGLPQ-------------    18
B7P5R2_IXOSC        ------------------------------------------------------------    0
B7P5Q9_IXOSC        --------------------MAPPWIRKTALVLLLACVVHNASAVVK----GSDA-----    31
A0A224YVR0_RHIZA    -MREVLRGFPDSFLRRLESVAITRRHGVSVLILLLAALQQQASAVTN----GRDA-----    50
L7MD51_RHIPU        GAREN-----TRFQQQYVAASXLPLLNNTLLTVHVSCLISRRLGKTH------DSSSN--    47
A0A293LT14_ORNER    ---------------------------MNL-----ILQLLCAAV----------------    12
A0A2R5LB07_ORNTU    ------------------------------------------------------------    0
V5ICA0_IXORI        ---------------------------MKIAILTLACLAHTVLGQSQRNGRQTNST----    29
D0E0G4_RHISA        ---------------------------MELLTLPLALLLTCALAMPQQNGSGSTSSTTST    33
A0A0A0QMB6_AMBAM    ---------------------------MKLIALPLALLLACALGMPQQDGDATTPSTSST    33

OeCHI               --------------------YAQDGAAVRQHRKKAVVCYWSSQSWKRPAPYNYSVQDIPA    60
B7P5Q8_IXOSC        QLLDSSGVLD------DRSA-SRLESPGDGTQRGRIVCYYSAWALYRPEPMNYDIEDIPL    64
B7QIK8_IXOSC        -----AENSS------AESA-VSKKVENGGGSRGTVVCYYQTWACKRPAPMKYDIEDIPV    55
A0A1Z5L6Y6_ORNMO    --------------------VLSSLPRYNHGRHVPVVCYVNKYTTTHPAPYNYHMGDIPI    58
B7P5R2_IXOSC        --------------------------------------------------MNYRIKDIPG    10
B7P5Q9_IXOSC        ---------------------TKGITIPRNKFLVPVVCYYYGWAIGRPPPMNYRVEDVPL    70
A0A224YVR0_RHIZA    ---------------------VEGVTIPRNKFEVPVVCYYYGWASQRPSPANYSIEDVPM    89
L7MD51_RHIPU        -MWRQA-------LMVLCAAHLLSAHADYEEDKKPVVCYYYGEAMTRPSPMNYRVSDIPG    99
A0A293LT14_ORNER    -----------AAASFAEN--ANAQGVRNRTSDLPIVCYYYGWASTRPHPMNYEPANLPG    59
A0A2R5LB07_ORNTU    ------------------N--AN-AQAANITSDLPVVCYYYGWANTRPHPMNYEPEDLPG    39
V5ICA0_IXORI        -IL------------------NVNSTAASSTHTMPIVCYYYGWAATRPDPASYDVDKIPG    70
D0E0G4_RHISA        -V------SGSSSTSAPANATTARTRSDTHLGPAPVVCYYYAWANARPHPANYGVKDIPG    86
A0A0A0QMB6_AMBAM    -SLSTSAASSVSTSQAPANATSSTTTGAASSGRSPVVCYYYGWANTRPNPANYGVDDIPG    92
                                                       :***       :* * .*   .:*

OeCHI               EQCSHLVYSSANIDTKTYNLTFQGSTVGANESVFDSAVGLKRKNPSLILLLRVGDCHHGY    120
B7P5Q8_IXOSC        DKCTHLIYAFVGLSNQTWELFSIDPEYDFNKGGYRRFTALRKKRYPKLQTLLAVGGWAEGG    124
B7QIK8_IXOSC        DLCSHLIYSFVGLDNKTWTVNNIDEDFDNKQDGLRRFVALKKKHPHLKTLLAIGGWDEGG    115
A0A1Z5L6Y6_ORNMO    HLCSHLVYSYGEIEENTWTVKDTLG-------TFSNATSLKEQSPRLKVLLSIGGWSAQS    111
B7P5R2_IXOSC        DLCTHVNLNYAGIDMDKLTVKDLIPAYQINPRQYKEFTGIKSKFLFVKTMISLGGWDQGG    70
B7P5Q9_IXOSC        DYCTFVVLAFAGIDDRTWELKSVIPEYQQNESLYRNFTDLKTQHLFLKTMLAVGGWEHGG    130
A0A224YVR0_RHIZA    DMCTFVVLAFVGIDNETFEIKSMIPEYEEDERKYLAFTDLKNRHLYLKTMLAVGGWDHGG    149
L7MD51_RHIPU        HLCNYVTLAFVTINSETFEIESSMSAYLNNTGLYEEFTSIKKKYSHIKTLVSVGGWEYGH    159
A0A293LT14_ORNER    DLCSHVNFAYAGVNAETFELKSEVPEYEADRALFKTFTSIKNKYPNLKTLLSIGGWQHEL    119
A0A2R5LB07_ORNTU    DLCTHVNFAFAGVDAETFELKSEVPQYEANRALFKKFTSIKARYPHLKTVLSIGGWQHNL    99
V5ICA0_IXORI        DLCTHVNFAYAGVAPNTWELDSQVPRYDADKGLYKKFTAIKEDYPKLKTLLSVGGWQHPN    130
D0E0G4_RHISA        DLCTHVNFAYAGVNPQTWELRSEVPEFESNRDLFKNFTAIKTQYPQLKTLLSVGGWQHEN    146
A0A0A0QMB6_AMBAM    DLCTHVNFAYAGVDPQTWELKSEVPEYEGNRELFKNFTAIKTRYTQLKTLLSVGGWQHEN    152
                    . *..:      :   .  :                 . ::     :  :: :*.
```

```
OeCHI               DPIASVLANSSEGRSAFAKSAFDWIVKHHFDGIALGWCKPKDN-------ERANLVKVVK   173
B7P5Q8_IXOSC        KKYS-EMVSTTGRRHTFINSALKWVQTYGFDGFDLDWEYPGAYDRGGAYSDKANFLLLVK   183
B7QIK8_IXOSC        KKYS-EMVSDKAKRKTFVKSALEWVLKYSFDGFDLDWEYPGASDRQGKYSDKENFLELVK   174
A0A1Z5L6Y6_ORNMO    NYIG-RIASQQGTRETFVQNILDTLKEHHFDGLDIFWQFPGFTERGGTPADKENFVLLAK   170
B7P5R2_IXOSC        EAFS-YVARDPVRRCNFTKNLYKFLKDNDFDGVDIDWRFPASPDRAGQPEDKENYVQLLK   129
B7P5Q9_IXOSC        KVFS-DMVSKPERRTMFVKSALDWMLRHGFDGLEIIWKYPGYEPRGGTSDDKDNFVQLLK   189
A0A224YVR0_RHIZA    EVFS-NMVSRPESRAAFVESAVRWMRDHEFDGLEILWKYPGYEKRGGKPRDKHNFVLLLK   208
L7MD51_RHIPU        SVFS-RMVSRTVSRRRFVKSAVRWMRHYGFDGLDISWQYPGTMVRGGSHADKENFVLVMK   218
A0A293LT14_ORNER    GSFT-LMVSSAHSRQKFVRSVLSWIDEYGFDGVDIDWQSPAVSERGGAPSDRRNYVLLVK   178
A0A2R5LB07_ORNTU    RSFS-PMVSSQATRQRFIRSLISWMDEYDFDGADMYWMYPAVPERGGAPTDRENYVQLLK   158
V5ICA0_IXORI        GVFS-QMSSTPQNRQKFITSVLQWMKEYNLDGVDLAWPFPGVDYRGGGPQDKENYVQLAK   189
D0E0G4_RHISA        GVFS-QLVASADRRALFIESVLLWMKEYNLDGVDMAWRFPGVSYRGGSPRDKENYASLIR   205
A0A0A0QMB6_AMBAM    GVFS-AMAANSNRRALFIESVLRWMKEYNLDGVDMAWPFPGVSYRGGSPRDKENYASLIR   211
                          :      *  *  .    :    :**  : *  *          :: *   : :

OeCHI               EVRKELSKVNRKTYILLEVPVDKKSLRTGYNVTELA-----RYVDYFHARTYSLRGSWVN   228
B7P5Q8_IXOSC        ELRSVFDQ--YKLLLTAAVPVAKFRLQEGYEVAELG-----ELLDWINVMTYDLRGNWAG   236
B7QIK8_IXOSC        EIRVAFDE--HKLLLTCAVPVAKFRLDEGYEVEQLAKQVEFELFDHIHVMSYDLRGNWAG   232
A0A1Z5L6Y6_ORNMO    EFREAARKKQEDYLLTATIPLSPYILDNGYDICRLS-----PYLDWMNAIAYELRGKWLN   225
B7P5R2_IXOSC        SFCS-LRK--KGLTVTATVPITPYYLDAGYDIKEMA-----KYVDWFNVLGFDLRGRWTG   181
B7P5Q9_IXOSC        ELKAAFNP--HPLFLTAAVPVEENYLEDGYDIPRLT-----RNVDWFNVLAYDLRGKWNR   242
A0A224YVR0_RHIZA    ELKEAFAK--YPLYLTATVPLDENYLDGGYDIANME-----RYVDWFNVLAYDLRGTWNR   261
L7MD51_RHIPU        DLKSEFQK--YDFMLSTTIPIDPMLLKTGFNVAALS-----RYVDWFNVFTHDLRGTWTG   271
A0A293LT14_ORNER    ELAAAFSN--RSLVLTITIPILPKYLDPGYDVAELS-----KYVAWFNVHTFDLRGRWNG   231
A0A2R5LB07_ORNTU    EISEVFRE--KKLLLTLEIPILPKYLDPGYDVAEMS-----KYVDWFNVHTFDLRGRWNG   211
V5ICA0_IXORI        ELSKELRE--NGLMLTMEVPLSDEHLNPGYDIDELA-----KYVDWFNVHAYDLRGKWNG   242
D0E0G4_RHISA        ELAGAFQG--KDLLLTVVVPVSDEFLDPGYNVAEIS-----KYVDWINAQAYDLRGAWNG   258
A0A0A0QMB6_AMBAM    ELAGAFEG--KGLLLTVVVPLPEEFLEAGYDIPEIS-----KHVDWINAQAYDLRGVWNG   264
                    ..            :   :*:    *  *:::  :        .  ::.  ..*** *

OeCHI               YTDVHSPLQKRPFEGNDLTDLNVKDGAQLLVKLGAPSQKILLGIPFFGTSYTLANATNHG   288
B7P5Q8_IXOSC        FTDVHSPLFRRSFDEWAYEKLNVHDGLQLWISLGAPREKLIVGVPFYGRTYTLSDKANTG   296
B7QIK8_IXOSC        FADVHSPLFKRPFDQWAYEKLNVDDGLKLWVSRGAPKHKLIVGIPLYGRTYTLGSKENHG   292
A0A1Z5L6Y6_ORNMO    QTDIHSPTVPRSIDSQDYKELNVKYGLRNLFDRCALKRKVMLGIAFYGRVYKLRSAGEHG   285
B7P5R2_IXOSC        IADVHSPLHARSFETGDVRNLNVERGLARLVELGAPKKKLVLGVPFFGRSFVLQDSNKTQ   241
B7P5Q9_IXOSC        FTDMHSILYKRAADSKYFQELTIAEGMKRLVRMGAPKDKLMLGIAFYGRTYVLRDPAKHG   302
A0A224YVR0_RHIZA    FTDVHSILYKRRNDPPYFQDLTIAQGMKRLVRMGAPKRKLMLGMGFYGRSYVLLDARDHG   321
L7MD51_RHIPU        YTDVHSPLRRRCFDRGQYAALNAEDGLNRLVKLGAPKKKLMLGIPFYGRSYTLLDPRRHS   331
A0A293LT14_ORNER    VTDVHSPLYSRSIDVGELKTLNVKDGLEKLVALGAPKSKLVVGIPFFGRQFTLLIPEQHE   291
A0A2R5LB07_ORNTU    VTDVHSPLYSRSIDVGEFKTLNVKDGMEKLVARGAPKKKLLMGIPFLGRQFTLLDEAQHG   271
V5ICA0_IXORI        VTDVHSPLFPRDIDVGDQQKLNVKDGLENLVSRGAPKSKLVMGIPFFGRGFTLLDRSQHG   302
D0E0G4_RHISA        VTDVHTPLFPRSIDFGPQATLNVKDGLARIVSLGAPKSKVVMGIAFFGRGFTLLDPQQHG   318
A0A0A0QMB6_AMBAM    VTDVHTPLYSRSIDIGPQKTLNVKDGLARIVSSGAPKSKVVMGIAFFGRGFTLLDPAQHG   324
                     :*:*:    *  :      *.   *    .   *   *:::*: : *  : *

OeCHI               LQAALRDKSPSGRPGQYTNHTGILAYFEICKAIAEHNLTRQWDATGLCPYAYSGDQWVGY   348
B7P5Q8_IXOSC        LRAYINKEKMGGIPGPYTNATGFLAYYEICPHVHSGTWTKKFDEVGKCPYAYYDNQWIGY   356
B7QIK8_IXOSC        LRAPIVQWVDGGTPGEFTNATGFQAYFEVCKNVKENGWTRRWDKDGRCPYAFKDNQWVGY   352
A0A1Z5L6Y6_ORNMO    LHAPIDTKT-EPLPGPALNTSEIYSYTEICQLLQMKNWTRNFDPEGKCPYAYSGDQWVGY   344
B7P5R2_IXOSC        VGAPIK-DA-PAVPGPFIGSTEIMAYYEICTNIVDEIATREFDKEAMCPYIHYDDQWIGY   299
B7P5Q9_IXOSC        VKARIKHDQ-PAEAGPYVRSHDLMGYYEICPNIKSGLWTRQFDQEAKCPYAYHGNQWVGY   361
A0A224YVR0_RHIZA    VKARIKFDE-QAEAGPYVKSTELKAYYEICMDLKKGGWTREFDDVAKCPYAYKGDQWIGY   380
L7MD51_RHIPU        LAAPTRRDV-HAVPGPYVMSDEILAYYEVCMDITMLSWRREYDEIGQCPYAYRGDQWVGY   390
A0A293LT14_ORNER    LHAIINPEA-TPSAGPFVRSTEILGYYEICLLLR-ASWIREFDNEGKCPYAYRRNMWVGY   349
A0A2R5LB07_ORNTU    LHAVINPSV-TPNEGAFVRSSEVMGYYEICLLLR-SSWIREFDTEGKCPYAYQKNMWVGY   329
V5ICA0_IXORI        LHAIINGNI-PPNPGPFVKSSDVYSYFEICLFLK-SGWTREFDDQGKAPYAYYNNQWIGY   360
D0E0G4_RHISA        LHALINREV-PPHAGPFVRSNEIFAYYEICLNLK-GNWKREFDDEGKCPYVYYRDQWIGY   376
A0A0A0QMB6_AMBAM    LHALVNRDV-PPRPGPFVRSNEVFAYYEICLNLK-GNWKREFDEEGKCPYVYYRDQWIGY   382
                    : *           *       . .* *:*  :      :.:*  . .** .  : *:**

OeCHI               EDEESIYHKARLIETENYGGAVVYTIDLDDFVGRCGRKHNLLKTVQSSLNPANVTVHVPV   408
B7P5Q8_IXOSC        EDEESITIKMDYIRGQGYGGAMIWAIDMDDFQGVCGKKNVLISAIHDKLKDYVVPTPESD   416
B7QIK8_IXOSC        EDEESVAIKVYWKRNS-YRLTMR-------------------------------------   374
A0A1Z5L6Y6_ORNMO    EDEESIQYKMDLMRDEGYAGVMVMSADMDDIRGLCGKNNILLETINDNLPKERQPIYPWT   404
B7P5R2_IXOSC        EDEESVGAKMDFIIKEGYAGVMVYNNDMDDFNGVCGKTHPLLKTIYEKLAELPER-RRR-   357
B7P5Q9_IXOSC        EDEESVANKMDFIIGQGYRGVMVFNNDLDDFRGVCGPKNPLMKVIFNKVGEKELR-ALNI   420
A0A224YVR0_RHIZA    EDEESVGYKMDFILREGYRGVMVFNNDLDDFRGLCGPKNPLMKVIFNKVGQKALL-ELKN   439
L7MD51_RHIPU        EDEESISAKVDFVLEQDYGGVMVFNIDMDDFNGVCGVKNPLLNSVCRKFNEGRL-IDPRI   449
A0A293LT14_ORNER    DDVDSIDYKTSFVTDEGYGGVHAFNVDLDDFRGICGTTNPLLKAINARLRPDQNAVE---   406
A0A2R5LB07_ORNTU    DDVDSIDYKTMFLIEAGYGGMHVFNVDLDDFRGICGATNPLLKAINARLRPSQNYVK---   386
V5ICA0_IXORI        EDTESIKHKMNFLLREGYAGVYVFNNDMDDFRGLCGEPNILLKTIRNSLNQDKNDIAETL   420
D0E0G4_RHISA        DDAESIQHKVDFLLQEGYRGVYVFNNDLDDFRGFCGEPNILLKTIKNGLTEKNE-LDARV   435
A0A0A0QMB6_AMBAM    DDAVSIRHKINFLLQEGYRGVYVFNNDLDDFRGFCGETNILLKTIKEGLNGNKNEITASP   442
                    :*  *:  *   :   .* *  .   *:**: * **  : *:. :   .
```

```
OeCHI               SAAHNVASKLPLALSYRFANYHGGATNGSNV-THPAARRNQT--NIYQAPTYKGDKCIN-    464
B7P5Q8_IXOSC        S-TAS---------------PKTGPDDGGNTTPEPTTPIPETTTPLSVTKSYPRD-CDSP    459
B7QIK8_IXOSC        ------------------------------------------------------------    374
A0A1Z5L6Y6_ORNMO    S-----------------------------------------------------------    405
B7P5R2_IXOSC        ------------------------------------------------------------    357
B7P5Q9_IXOSC        NPT---------------------------------------------------------    423
A0A224YVR0_RHIZA    RTGSQ---------------QPEGTTTRGNVLV---------------------------    457
L7MD51_RHIPU        G-----------------------------------------------------------    450
A0A293LT14_ORNER    ------------------------------------------------------------    406
A0A2R5LB07_ORNTU    ------------------------------------------------------------    386
V5ICA0_IXORI        ------------------------------------------------------------    420
D0E0G4_RHISA        NEKV--------------------------------------------------------    439
A0A0A0QMB6_AMBAM    A-----------------------------------------------------------    443

OeCHI               GMCITAGSTVPTLSVFVTVFA--AVLFSRL------------------------------    492
B7P5Q8_IXOSC        NISFIPHE-NDCTKYYWCVYGTPMVMFCEGGTVWNQDNGNCDWPERVPRPECKHVTRKPK    518
B7QIK8_IXOSC        ------------------------------------------------------------    374
A0A1Z5L6Y6_ORNMO    ------------------------------------------------------------    405
B7P5R2_IXOSC        ------------------------------------------------------------    357
B7P5Q9_IXOSC        ------------------------------------------------------------    423
A0A224YVR0_RHIZA    ------------------------------------------------------------    457
L7MD51_RHIPU        ------------------------------------------------------------    450
A0A293LT14_ORNER    ------------------------------------------------------------    406
A0A2R5LB07_ORNTU    ------------------------------------------------------------    386
V5ICA0_IXORI        ------------------------------------------------------------    420
D0E0G4_RHISA        ------------------------------------------------------------    439
A0A0A0QMB6_AMBAM    ------------------------------------------------------------    443

                                         % Identity
OeCHI               --------  492        -
B7P5Q8_IXOSC        GPQRLESS  526        38.61
B7QIK8_IXOSC        --------  374        41.09
A0A1Z5L6Y6_ORNMO    --------  405        37.50
B7P5R2_IXOSC        --------  357        35.41
B7P5Q9_IXOSC        --------  423        36.50
A0A224YVR0_RHIZA    --------  457        37.00
L7MD51_RHIPU        --------  450        38.04
A0A293LT14_ORNER    --------  406        37.10
A0A2R5LB07_ORNTU    --------  386        36.00
V5ICA0_IXORI        --------  420        37.30
D0E0G4_RHISA        --------  439        36.27
A0A0A0QMB6_AMBAM    --------  443        35.37
```

b

OeCHI

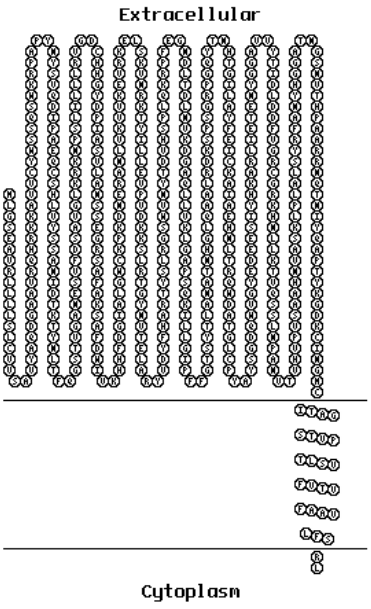

Signal P	1	22
Outside	23	467
**TM helix**	**468**	**490**
Inside	491	492

c

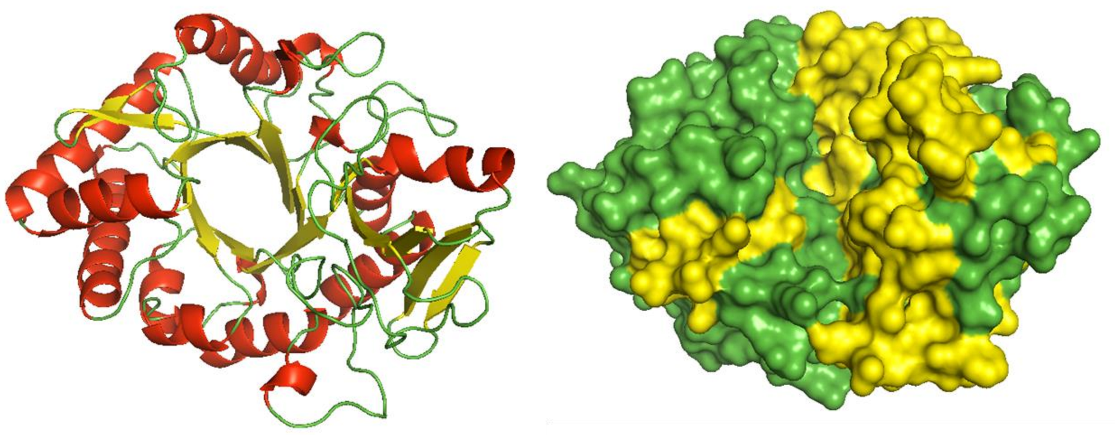

Supplement: Supplementary file 2 — Additional file 2: Figure S1. Tick chitinases sequence alignment and OeCHI topology prediction. [file 13071_2019_3768_MOESM2_ESM.pdf]
