# Supplementary material for: In silico selection of functionally important proteins from the mialome of Ornithodoros erraticus ticks and assessment of their protective efficacy as vaccine targets
Source: Parasit Vectors. 2019 Oct 30;12:508. doi: 10.1186/s13071-019-3768-1 (PMC6822432; doi:10.1186/s13071-019-3768-1)

Additional file 3: Figure S2. a, b Multiple alignments of the amino acid sequences of A0A293MYE4_ORNER (OeTSP1) and A0A293M0B7_ORNER (OeTSP2) with their respective tick orthologues. **c** Pairwise alignment of OeTSP1 and OeTSP2. Uniprot entry names are shown: AMBAU, *Amblyomma aureolatum*; AMBSC, *A. sculptum*; AMBTR, *A. triste*; CARMI, *Carios mimon*; IXORI, *Ixodes ricinus*; IXOSC, *I. scapularis*; ORNER, *Ornithodoros erraticus*; ORNMO, *O. moubata*; ORNTU, *O. turicata*. The amino acids conserved in all sequences are labelled with asterisks (*); the conservative and semi-conservative substitutions are labelled with colons (:) and periods (.), respectively. The percentage of sequence identity showed by OeTSP1 and OeTSP2 to their orthologues in each alignment is indicated in the column on the lower right corner. **d** Topology predictions for OeTSP1 and OeTSP2 using the TMHMM server. The values indicate the amino acids forming part of each region. **e** Three dimensional modelling. Left: 3D models of both TSPs showing the typical 3D structure for monomeric Tetraspanin (TM4SF) family members. Right: 3D models showing the predicted linear B-cell epitopes (yellow) covering most part of the surface of the long extracellular exposed domains.

a

```
Q6QSX5_ORNMO        MDDGIACVKYVLIACNLLVWILGLAVLSIGIWIRSDPDFWIYQDNLPLSNYYDACYIIMA    60
A0A293MYE4_ORNER    MDGGIACVKYVLIACNLLVWILGLGVLSIGIWIRSDPDFWIYQDNLPLSNYYDACYVIMA    60
A0A2R5L5I4_ORNTU    MDDGIACVKYVLIACNLLVWMLGLGVLSIGIWIRSDPDFWVYQDNLPLSNYYDACYVVMA    60
A0A147B935_CARMI    --EGVACIKYVLIACNLLVWILGLGVLSIGIWIRSDPDFWIYQDNLPLSNYYDACYVVMA    58
V5ICR4_IXORI        MDEGIACVKYILITCNLLVWMLGLGVLAVGIWIRSDPDFWVYQDNLPLSNYYSACYVIIA    60
V5HK55_IXORI        ----------------------------------RSDPDFWVYQDNLPLSNYYSACYVIIA    27
A0A023GHE1_AMBTR    MDEGVACVKYILITCNLFVWILGLGVLSVGIWIRSDPDFWVYQDNLPLSNYYSACYVVMA    60
A0A1E1X3I4_AMBAU    MDEGVACVKYILITCNLFVWILGLGVLSVGIWIRSDPDFWVYQDNLPLSNYYSACYVVMA    60
A0A1E1XRP3_AMBSC    MDEGVACVKYILITCNLFVWMLGLGVLSVGIWIRSDPDFWVYQDNLPLSNYYSACYVVMA    60
                    **.*:**:**:**:***:**:***.**::***********:***********.***::*

Q6QSX5_ORNMO        AGVLLLILGFMGCCAAAIDSPCMLLTYFIAMLVLLLMECAVAGLVWKVADGDTLQRHLAT    120
A0A293MYE4_ORNER    VGVLLLVLGFMGCCAAAIDSPCMLLTYFIAMLALLLMECAVAGLVWKVADGDTLQRYLAT    120
A0A2R5L5I4_ORNTU    VGVLLLILGFMGCCAAAIDSPCMLLTYFIAMLVLLLMECAVAGLVWKVADGDTLQRHLAT    120
A0A147B935_CARMI    VGVLLLILGFMGCCAAAIDSPCMLLTYFIAMLVLLLMECAVAGLVWKVADGDTLQRHLAS    118
V5ICR4_IXORI        VGVLLLVLGFMGCCAAAIDSPCMLLTYFIAMFVLLILECAVAGLVWKVADGDQLQQKLAE    120
V5HK55_IXORI        VGVLLLVLGFMGCCAAAIDSPCMLLTYFIAMFVLLILECAVAGLVWKVADGDQLQQKLAE    87
A0A023GHE1_AMBTR    VGVLLLVLGFMGCCAAAIDSPCMLLTYFIAMFVLLIMECAVAGLVWKVADGDQLQHHLAV    120
A0A1E1X3I4_AMBAU    VGVLLLVLGFMGCCAAAIDSPCMLLTYFIAMFVLLVMECAVAGLVWKVADGDQLQRHLAV    120
A0A1E1XRP3_AMBSC    VGVLLLVLGFMGCCAAAIDSPCMLLTYFIAMFVLLIMECAVAGLVWKVADGDQFQRHLAV    120
                    .*****:************************:.**::*************** :*: **

Q6QSX5_ORNMO        TITAKIDEINDNPKARRFMDLMQVHLECCGAISKHDYEVRAMTIPQSCSSSRTNNIFIYG    180
A0A293MYE4_ORNER    TITEKIDEINENPKTRRFMDLMQVHLECCGAISKHDYEVRAMTIPQSCSSSRTNNIFIYG    180
A0A2R5L5I4_ORNTU    TIMERIDEINEDPKARRFMDLMQVHLECCGAISKHDYEVRAMTIPQSCSSSRTNNIFIYG    180
A0A147B935_CARMI    TIMERIDEINDNPKTRRFMDLMQVHLECCGAISKHDYEVRAMTIPQSCSSSRTNNIFIYG    178
V5ICR4_IXORI        SIGQKMDTIIEDSKARRFMDLMQVHLECCGAISKHDYEVRSLTIPQSCSSSRTNNIFYYG    180
V5HK55_IXORI        SIGQKMDTIIDDSKARRFMDLMQVHLECCGAISKHDYEVRSLTIPQSCSSSRTNNIFYYG    147
A0A023GHE1_AMBTR    AIEQKLDTVVDDPKARRFMDLMQVHLECCGAISKHDYETRDLTIPQSCSSSRTNNIFIYG    180
A0A1E1X3I4_AMBAU    AIEEKLDTIVDDSKARRFMDLMQVHLECCGAISKHDYEVRDLTIPQSCSSSRTNNIFIYG    180
A0A1E1XRP3_AMBSC    AIEEKLDTIVDDSKARRFMDLMQVHLECCGAISKRDYDVRDLTIPQSCSSSRTNNIFIYG    180
                    :*   ::* : ::  *:*******************:**:.*  :*************** **

                                                                          % Identity
Q6QSX5_ORNMO        CSENLRVLLERTGAVVGGMGLALGFVQVIVMIISLCLFCTIRQDSK  226           94.2
A0A293MYE4_ORNER    CSENLRVLLERTGAVVGGMGLALGFVQIIVMIISLCLFCTLRQDGK  226           -
A0A2R5L5I4_ORNTU    CSENLRVLLERTGAVVGGMGLALGFVQIIVMIISLCLFCTLRQDSK  226           94.7
A0A147B935_CARMI    CSENLRVLLERTGAVVGGMGLALGFVQIVVMIISLCLFCTLRQDSK  224           94.6
V5ICR4_IXORI        CSENLRVLLERTGAVVGGLGLALGFVQIIVMIISLCLFCTIRQDSK  226           85.8
V5HK55_IXORI        CSENLRVLLERTGAVVGGLGLALGFVQIIVMIISLCLFCTIRQDSK  193           86.0
A0A023GHE1_AMBTR    CSENLRVLLERTGAVVGGLGLALGFVQIIVMIISLCLFCTIRQDSK  226           85.8
A0A1E1X3I4_AMBAU    CSENLRVLLERTGAVVGGLGLALGFVQIIVMIMSICLFCTIRQDTK  226           86.3
A0A1E1XRP3_AMBSC    CSENLRVLLERTGAVVGGLGLALGFVQIIVMIISLCLFCTIRQDSK  226           85.4
                    *****************:********::***:*:*****:*** *
```

b

```
B7Q3S5_IXOSC        -----------------------------MGSTVLRYCLFFSSLAVWLFGVALLVLGGLL    31
A0A2R5LCA7_ORNTU    -----------------------------MGYRWLRFTLITTNVVVWLLGLSLLVAGCLL    31
A0A293M2J9_ORNER    MHAVVRHVRREFADGGGSHEVTVDTGPTEMGYRWLRYTLFATNVIVWLLGLALLVIGCLL    60
A0A293M0B7_ORNER    ----MADEEAQRTTGGGS----GYKRPPKSQSKCIKFTLIATNSVIWLLGLTVFILSVVL    52
A0A147B7T2_CARMI    ------------------------------------------------------------    0
V5HFA0_IXORI        ------------------------------------------------------------    0
A0A131Y6A5_IXORI    ------------------------------------------------------------    0
A0A023G3M7_AMBTR    ------------------------------------------------------------    0
A0A1E1XRJ4_AMBSC    ------------------------------GLTCVKYCLFIVNLAVWLLGLAVIVVSAVA    30
A0A1E1XD95_AMBAU    ------------------------------GFTCVKYCLFVVNLAVWLLGLAVIVVSAVA    30
                                                  ::: *:   .   :**:*::::: . :
```

```
B7Q3S5_IXOSC        AADLNVRQVREFFDVFDNYGAASILTLVVGSCLFCIGFLGCCGSFFKDSLLLIPYNVLML   91
A0A2R5LCA7_ORNTU    IVDQDVKEVTHNFDIFDNYRVAGVFTIVVGTCLSCVGFLGCCGALFLNGCLLLTYIMLMA   91
A0A293M2J9_ORNER    VVDKDVKEVTQNFDIFDNYRVAAVFTLVLGICLFCVGFLGCCGALFLNWFLLLAYIMSMA   120
A0A293M0B7_ORNER    ICAR-HT--STELDAVQSLRDAAITGIVLGLILFCTGFLGCCGAAKENVCMLATYFIILA   109
A0A147B7T2_CARMI    --GK-GS--SSDLDAIRSLRNSAITGICLGLVLFWTGFLGCCGAAKENVCMLATYVVILV   55
V5HFA0_IXORI        ------------------------------------------------------YFGVLL   6
A0A131Y6A5_IXORI    ------------------------------------------------------YFGVLL   6
A0A023G3M7_AMBTR    ------E--GAQLAGLQSVRSAAATGLFIGCLLFLVGFLGCCGAMRESRVMLTCYFVILL   52
A0A1E1XRJ4_AMBSC    LAGAPTD--EATLAGLQSVRSAAATGLFIGCLLFLVGFLGCCGSMRESPAMLTGYFVILL   88
A0A1E1XD95_AMBAU    LAGAPSD--EASLAGLQSVRSSAATGLFIGCLLFLVGFLGCCGSMRESSVMLTGYFIIML   88
                                :  .:.   :.   :.:*  *   *******:   .  :*  * : :

B7Q3S5_IXOSC        LFIILELTVMGLVWKHANGEQLENNLSEAFVKLIVKSKSGLAE-----VERFVDNIQYRL   146
A0A2R5LCA7_ORNTU    VFLVLEFTVMGLVWKHANVYELEENVSEAFRKLILKSRNGIFA-----VEMFLDRVQQDL   146
A0A293M2J9_ORNER    VFVVLEFTVMGLVWKHANVHELEEQVSEAFRKLILKSRNGVYV-----VERFLDRLQQDL   175
A0A293M0B7_ORNER    VALIIEIAVMALALAYVSTSKLDDIVTVAFDQMIKGGSRE--------DTELLYSTQQNM   161
A0A147B7T2_CARMI    VAMLFEIAVMALALTYASSSKLDENITRIMKEMVTGGDRH--------EAELLYQLQEDL   107
V5HFA0_IXORI        VILVFEIVAIALAFSFVNSSSMEQSLNDHFVDVISGGRREKEPWKQEEDLNVIYFVQGQL   66
A0A131Y6A5_IXORI    VILVFEIVAIALAFSFVNSSSMEQSLNDHFADVISGGRREKEPWKQEEDLNVIYFVQGQL   66
A0A023G3M7_AMBTR    VIAIFLIAVMALAFSYINSSKMEEALSEHFKDVITGGRRDKEPWQQEEDKEAVLFVQTEF   112
A0A1E1XRJ4_AMBSC    VIAIFLIAVMALAFSYVNSSTMENALNEHFKDVITGGRRDKDPKAQEEDMDTILFVQAEF   148
A0A1E1XD95_AMBAU    VIAIFLVAVMALAFSFVNSSKMEEALSEHFKDVITGGRREKDPKEQEKDMESVLYLQTEF   148
                    :  :: ...:.*.  . .   ::: :.  : .::  .               :   *  :

B7Q3S5_IXOSC        QCCGGLGPKDYEALEMPPTTSCKYYDSNEEQAAAYQQGCGRAIRNFLMSKSLAIGLVCLI   206
A0A2R5LCA7_ORNTU    KCCGGHGPDDYILLEMDCSAGCFYYTSGE--VVTYSQGCGKAVSDFLMGKSLAIGLVCLF   204
A0A293M2J9_ORNER    ECCGGRGPDDYTLLEMDRTAGCFYYASSE--VLAYSKGCGEAVSDFLMRKSLAIGLVCLF   233
A0A293M0B7_ORNER    RCCGATGIDDYAGHDMPVPPSCFDS-SEDNRSYIYADGCVTALKHYLRSNGLSIGLIAFF   220
A0A147B7T2_CARMI    RCCGGSGPNDYTDESLSLPPSCYDD-TSFGGPYMFQEGCVSALKRYLSSNGLAIGLVSLF   166
V5HFA0_IXORI        RCCGGKGPEDYAERHLPIPPSCYDN-YDTQRTYIYQRGCVAALKEYVRKNGLSIGLVNMF   125
A0A131Y6A5_IXORI    RCCVGKGPEDYSERHLPIPPSCYDN-YDTQRTYIYQRGCVAALKEYVRKNGLSIGLVNMF   125
A0A023G3M7_AMBTR    RCCGGRGPQDYVDNNLDVPPSCYDT-QDS-------------------------------   140
A0A1E1XRJ4_AMBSC    RCCGGRGPQDYTEASAAIPPSCYDS-RDVQRPYLFQTGCSKAMQKYILRNGLGLGLISLF   207
A0A1E1XD95_AMBAU    RCCGGRGPQDYIDSGVAVPPSCYDA-RDSQRPYLFQTGCSKAMQKYILRNGLGLGLTSLF   207
                    .***. * .**         .*            :  **  *: .::  :.*.:** .::

                                                                % Identity
B7Q3S5_IXOSC        VLLIEVFSVASAIYLLVEKKKKNRPKVTPV--       236      30.3
A0A2R5LCA7_ORNTU    IILIQLFAVGCAVYLYLDQRNKKPTPV-----       231      33.6
A0A293M2J9_ORNER    ILLLELFAVGSAVYLYLDKRKKKATPV-----       260      31.2
A0A293M0B7_ORNER    TFFAQVCSMAGAAVIIKRKPRGKLTP------       246      -
A0A147B7T2_CARMI    IFFIQVCSMACAVTIMRSKRSGKLPP------       192      58.2
V5HFA0_IXORI        GVFAEIAAMVGACMLMQHFKSAKKAGKNTTQA       157      41.6
A0A131Y6A5_IXORI    GVFAEIAAMVGACMLMQHFKSAKKAGKNTTQA       157      40.3
A0A023G3M7_AMBTR    --------------------------------       140      44.1
A0A1E1XRJ4_AMBSC    TLLALIIAMVCSCLLIQGIKKGRQLHTSVA--       237      39.2
A0A1E1XD95_AMBAU    TLFALIIAMICSCLLIQGIKKNRQLHTSVA--       237      39.4
                     ::  : ::  :  :     . .
```

C

```
A0A293MYE4 OeTSP1   -------------------MDGGIACVKYVLIACNLLVWILGLGVLSIGIWIRSDPDFWI   41
A0A293M0B7 OeTSP2   MADEEAQRTTGGGSGYKRPPKSQSKCIKFTLIATNSVIWLLGLTVFILSVVLICARHTST   60
                                        ..   *:*:.*** * ::*:*** *: :.: : .  .

A0A293MYE4 OeTSP1   YQDNLPLSNYYDACYVIMAVGVLLLVLGFMGCCAAAIDSPCMLLTYFIAMLALLLMECAV   101
A0A293M0B7 OeTSP2   --ELDAVQSLRDAAITGIVLGLILFCTGFLGCCGAAKENVCMLATYFIILAVALIIEIAV   118
                      :   :..  **. . :.:*::*:  **:***.** :. *** **** : . *::* **

A0A293MYE4 OeTSP1   AGLVWKVADGDTLQRYLATTITEKIDEINENPKTRRFMDLMQVHLECCGAISKHDYEVRA   161
A0A293M0B7 OeTSP2   MALALAYVSTSKLDDIVTVAFDQMIKG--GSREDTELLYSTQQNMRCCGATGIDDYAGHD   176
                     .*.   .. ..*:  ::.:: : *.    . :  .::   * ::.**** . .**  :

A0A293MYE4 OeTSP1   MTIPQSCSSSRTN---NIFIYGCSENLRVLLERTGAVVGGMGLALGFVQIIVMIISLCLF   218
A0A293M0B7 OeTSP1   MPVPPSCFDSSEDNRSYIYADGCVTALKHYLRSNGLSIGLI-------AFFTFFAQVCSM   229
                    * :* ** .*  :    *:  **   *:  *. .*  :* :        ::.:: .:* :

A0A293MYE4 OeTSP1   C------TLRQDGK---  226       27.3% identity
A0A293M0B7 OeTSP2   AGAAVIIKRKPRGKLTP  246
                    .      . :  **
```

d

OeTSP1

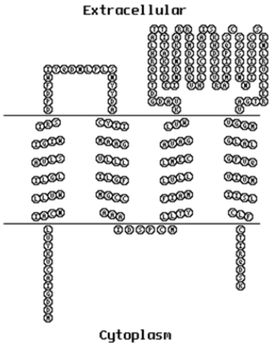

inside	1	12
**TMhelix**	**13**	**35**
outside	36	54
**TMhelix**	**55**	**77**
inside	78	83
**TMhelix**	**84**	**106**
outside	107	195
**TMhelix**	**196**	**218**
inside	219	226

OeTSP2

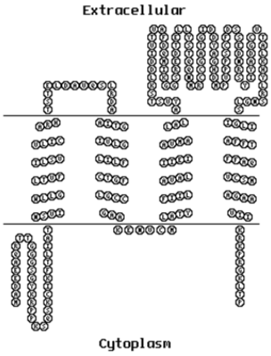

inside	1	34
**TMhelix**	**35**	**57**
outside	58	71
**TMhelix**	**72**	**94**
inside	95	100
**TMhelix**	**101**	**123**
outside	124	213
**TMhelix**	**214**	**236**
inside	237	246

e

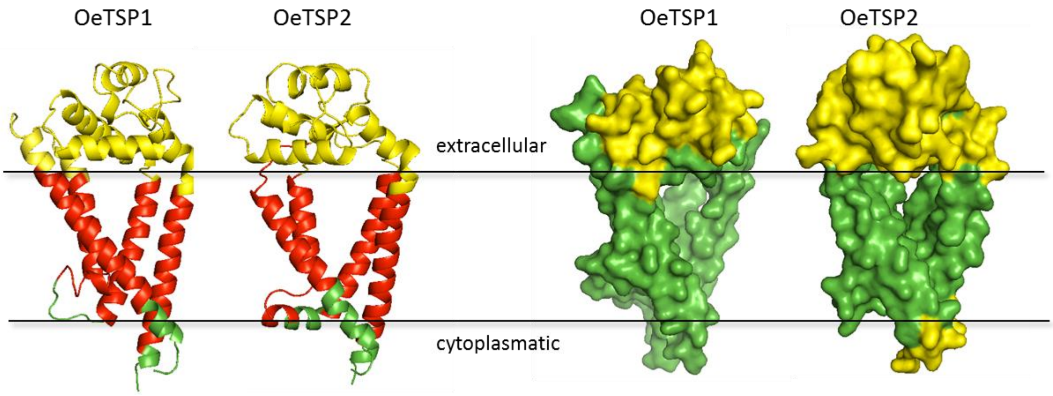

Supplement: Supplementary file 3 — Additional file 3: Figure S2. Tick tetraspanins sequence alignment and OeTSPs topology prediction. [file 13071_2019_3768_MOESM3_ESM.pdf]
