# Supplementary material for: In silico selection of functionally important proteins from the mialome of Ornithodoros erraticus ticks and assessment of their protective efficacy as vaccine targets
Source: Parasit Vectors. 2019 Oct 30;12:508. doi: 10.1186/s13071-019-3768-1 (PMC6822432; doi:10.1186/s13071-019-3768-1)

Additional file 4: Figure S3. a Alignment of the amino acid sequences of RPP0 and its tick orthologues. OeRPP0, 60S acidic ribosomal protein P0 from *Ornithodoros erraticus*. For the other sequences their Uniprot entry names are shown: AMBAM, *Amblyomma americanum*; AMBCA, *A. cajennense*; AMBMA, *A. maculatum;* AMBTR; *A. triste*; DERNI, *Dermacentor nitens*; HAELO, *Haemaphysalis longicornis*; HYAEX, *Hyalomma excavatum*; IXORI, *Ixodes ricinus*; IXOSC, *I. scapularis*; ORNER, *Ornithodoros erraticus*; ORNMO, *O. moubata*; ORNTU, *O. turicata*; RHIHA, *Rhipicephalus haemaphysaloides*; RHIMP, *R. microplus*; RHIPU, *R. pulchellus*; RHISA, *R. sanguineus*; RHIZA, *R. zambeziensis*. The amino acids conserved in all sequences are labelled with asterisks (*); the conservative and semi-conservative substitutions are labelled with colons (:) and periods (.), respectively. The percentage of sequence identity showed by OeRPP0 and its orthologues in the alignment is indicated in the column on the lower right corner. The 8 amino acid residues that form the 28S rRNA interface are highlighted in yellow and the 20 residues that constitute the putative interface with the L7/L12 ribosomal proteins are in blue. The conserved protective synthetic peptide by Rodriguez-Mallon et al. [42] is highlighted in grey. **b** 3D model of the OeRPP0. Left: conserved secondary structure of RPP0s. Right: the linear B-cell epitope predictions on the OeRRP0 surface (yellow). In purple, the residues of the interface with the L7/L12 ribosomal proteins that do not form part of any predicted epitope.

a

```
OeRPP0               MVREDKTTWKSNYLLRLVQLLDEYPKCFIVGVDNVGSKQMQTIRVSLRSHASLLMGKNTM   60
A0A1Z5LG66_ORNMO     MVREDKATWKSNYFLRLVQLLDEYPKCFIVGVDNVGSKQMQTIRVSLRSHASLLMGKNTM   60
A0A2R5L843_ORNTU     MVREDKTTWKSNYFVRLVQLLDEYPKCFIVGVDNVGSKQMQTIRVSLRNHACLLMGKNTM   60
A0A131XRJ4_IXORI     MVREDKTAWKSNYFLRLVQLLDEYPKCFIVGVDNVGSKQMQTIRVSLRKHAVLLMGKNTM   60
A0A0K8REE7_IXORI     MVREDKTAWKSNYFLRLVQLLDEYPKCFIVGVDNVGSKQMQTIRVSLRKHAVLLMGKNTM   60
B7PRG2_IXOSC         MVREDKTAWKSNYFLRLVQLLDEYPKCFIVGVDNVGSKQMQTIRVSLRKHAVLLMGKNTM   60
Q4PMB4_IXOSC         MVREDKTAWKSNYFLRLVQLLDEYPKCFIVGVDNVGSKQMQTIRVSLRKHAVLLMGKNTM   60
A0A0C5Q1J9_DERNI     MVREDKTTWKSNYFLRLVQLFDEYPKCFIVGVDNVGSKQMQTIRVSLRKHAVLLMGKNTM   60
A0A131XM65_HYAEX     MVREDKTTWKSNYFLRLVQLLDEYPKCFIVGVDNVGSKQMQTIRVSLRKHAVLLMGKNTM   60
A0A0C5PNS0_RHISA     MVREDKTTWRSNYFLRLVQLLDEYPKCFIVGVDNVGSKQMQTIRVSLRKHAVLLMGKNTM   60
A0A0P0D331_RHIHA     MVREDKTTWKSNYFLRLVQLLDEYPKCFIVGVDNVGSKQMQTIRVSLRKHAVLLMGKNTM   60
A0A224YGD4_RHIZA     MVREDKTTWKSNYFLRLVQLLDEYPKCFIVGVDNVGSKQMQTIRVSLRKHAVLLMGKNTM   60
S5FVY5_RHIMP         MVREDKTTWKSNYFLRLVQLLDEYPKCFIVGVDNVGSKQMQTIRVSLRKHAVLLMGKNTM   60
L7MM43_RHIPU         MVREDKTTWKSNYFLRLVQLLDEYPKCFIVGVDNVGSKQMQTIRVSLRKHAVLLMGKNTM   60
A9Y1V1_HAELO         MVREDKTTWKSNYFLRLVQLLDEFPKCFIVGVDNVGSKQMQTIRVSLRKHAVLLMGKNTM   60
A0A023FKN1_AMBCA     MVREDKTTWKSNYFLRLVQLLDEFPKCFIVGVDNVGSKQMQTIRVSLRKHAVLLMGKNTM   60
A0A023FIA8_AMBCA     MVREDKTTWKSNYFLRLVQLLDEFPKCFIVGVDNVGSKQMQTIRVSLRKHAVLLMGKNTM   60
A0A0C5PTI6_AMBCA     MVREDKTTWKSNYFLRLVQLLDEFPKCFIVGVDNVGSKQMQTIRVSLRKHAVLLMGKNTM   60
A0A0C9R2E6_AMBAM     MVREDKTTWKSNYFLRLVQLLDEFPKCFIVGVDNVGSKQMQTIRVSLRKHAVLLMGKNTM   60
G3MMD6_AMBMA         MVREDKTTWKSNYFLRLVQLLDEFPKCFIVGVDNVGSKQMQTIRVSLRKHAVLLMGKNTM   60
A0A023GIB1_AMBTR     MVREDKTTWKSNYFLRLVQLLDEFPKCFIVGVDNVGSKQMQTIRVSLRKHAVLLMGKNTM   60
                     ******::*:***::*****:**:************************.** ********

OeRPP0               IRKAIRGHLENNPALEKLLPHIKGNVGFVFTKEDLTDVREKILENKVKAPARAGALAPLD   120
A0A1Z5LG66_ORNMO     IRKAIRGHLENNPALEKLLPHIKGNVGFVFTKEDLTDVREKILENKVKAPARAGALAPLD   120
A0A2R5L843_ORNTU     IRKAIRGHLESNPALEKLLPHIKGNVGFVFTKEDLTEVREKILDNKVKAPARAGALAPLD   120
A0A131XRJ4_IXORI     IRKAIRGHLDNNPALEKLLPHIKGNVGFVFTKEDLGEVREKIIENKVKAPARAGALAPLD   120
A0A0K8REE7_IXORI     IRKAIRGHLDNNPALEKLLPHIKGNVGFVFTKEDLGEVREKIIENKVKAPARAGALAPLD   120
B7PRG2_IXOSC         IRKAIRGHLDNNPALEKLLPHIKGNVGFVFTKEDLGEVREKIIENKVKAPARAGALAPLD   120
Q4PMB4_IXOSC         IRKAIRGHLDNNPALEKLLPHIKGNVGFVFTKEDLGEVREKIIENKVKAPARAGALAPLD   120
A0A0C5Q1J9_DERNI     IRKAIRGHLDNNPALEKLLPHIKGNVGFVFTKEDLTEVREKIIDNKVKAPARAGALAPLD   120
A0A131XM65_HYAEX     IRKAIRGHLDNNPALEKLLPHIKGNVGFVFTKEDLTEAREKIIENKVKAPARAGALAPLD   120
A0A0C5PNS0_RHISA     IRKAIRGHLDNNPALEKLLPHIKGNVGFVFTKEDLTEVREKIIDNKVKAPARAGALAPLD   120
A0A0P0D331_RHIHE     IRKAIRGHLDNNPALEKLLPHIKGNVGFVFTKEDLTEVREKIIDNKVKAPARAGALAPLD   120
A0A224YGD4_RHIZA     IRKAIRGHLDNNPALEKLLPHIKGNVGFVFTKEDLTEVREKIIDNKVKAPARAGALAPLD   120
S5FVY5_RHIMP         IRKAIRGHLDNNPALEKLLPHIKGNVGFVFTKEDLTEVREKIIDNKVKAPARAGALAPLD   120
L7MM43_RHIPU         IRKAIRGHLDNNPALEKLLPHIKGNVGFVFTKEDLTEVREKIIDNKVKAPARAGALAPLD   120
A9Y1V1_HAELO         IRKAIRGHLDNNPALERLLPHIKGNVGFVFTKEDLTEVREKIIENKVKAPARAGALAPLD   120
A0A023FKN1_AMBCA     IRKAIRGHLDNNPALEKLLPHIKGNVGFVFTKEDLTEVREKIIENKVKAPARAGALAPLD   120
A0A023FIA8_AMBCA     IRKAIRGHLDNNPALEKLLPHIKGNVGFVFTKEDLTDVREKIIENKVKAPARAGALAPLD   120
A0A0C5PTI6_AMBCA     IRKAIRGHLDNNPALEKLLPHIKGNVGFVFTKEDLTEVREKIIENKVKAPARAGALAPLD   120
A0A0C9R2E6_AMBAM     IRKAIRGHLDNNPALEKLPPHIKGNVGFVFTKEDLTEVREKIIENKVKAPARAGALAPLD   120
G3MMD6_AMBMA         IRKAIRGHLDNNPALEKLLPHIKGNVGFVFTKEDLTEVREKIIENKVKAPARAGALAPLD   120
A0A023GIB1_AMBTR     IRKAIRGHLDNNPALEKLLPHIKGNVGFVFTKEDLTEVREKIIENKVKAPARAGALAPLD   120
                     *********:.*****:* **************** :.****::****************
```

```
OeRPP0              VWIPPQNTGLGPEKTSFFQALQIPTKIAKGTIEILNEIHLIKKDDRVGASEATLLNMLNI   180
A0A1Z5LG66_ORNMO    VWIPPQNTGLGPEKTSFFQALQIPTKIAKGTIEILNEIHLIKKDDRVGASEATLLNMLNI   180
A0A2R5L843_ORNTU    VMIPPQNTGLGPEKTSFFQALQIPTKIAKGTIEILNEIHLIKKDDRVGASEATLLNMLNI   180
A0A131XRJ4_IXORI    VMIPAQNTGLGPEKTSFFQALQIPTKISKGTIEILNEIHLIKKDDRVGASEATLLNMLNI   180
A0A0K8REE7_IXORI    VMIPAQNTGLGPEKTSFFQALQIPTKISKGTIEILNEIHLIKKDDRVGASEATLLNMLNI   180
B7PRG2_IXOSC        VMIPAQNTGLGPEKTSFFQALQIPTKISKGTIEILNEIHLIKKDDRVGASEATLLNMLNI   180
Q4PMB4_IXOSC        VMIPAQNTGLGPEKTSFFQALQIPTKISKGTIEILNEIHLIKKDDRVGASEATLLNMLNI   180
A0A0C5Q1J9_DERNI    VMIPAQNTGLGPEKTSFFQALQIPTKISKGTIEILNEIHLIKKDDRVGASEATLLNMLNI   180
A0A131XM65_HYAEX    VMIPAQNTGLGPEKTSFFQALQIPTKISKGTIEILNEIHLIKKDDRVGASEATLLNMLNI   180
A0A0C5PNS0_RHISA    VMIPAQNTGLGPEKTSFFQALQIPTKISKGTIEILNEIHLIKKDDRVGASEATLLNMLNI   180
A0A0P0D331_RHIHE    VMIPAQNTGLGPEKTSFFQALRIPTKISKGTIEILNEIHLIKKDDRVGASEATLLNMLNI   180
A0A224YGD4_RHIZA    VMIPAQNTGLGPEKTSFFQALQIPTKISKGTIEILNEIHLIKKDDRVGASEATLLNMLNI   180
S5FVY5_RHIMP        VMIPAQNTGLGPEKTSFFQALQIPTKISKGTIEILNEIHLIKKDDRVGASEATLLNMLNI   180
L7MM43_RHIPU        VMIPAQNTGLGPEKTSFFQALQIPTKISKGTIEILNEIHLIKKDDRVGASEATLLNMLNI   180
A9Y1V1_HAELO        VMIPAQNTGLGPEKTSFFQALQIPTKISKGTIEILNEIHLIKKDDRVGASEATLLNMLNI   180
A0A023FKN1_AMBCA    VMIPAQNTGLGPEKTSFFQALQIPTKISKGTIEILNEIHLIKKDDRVGASEATLLNMLNI   180
A0A023FIA8_AMBCA    VMIPAQNTGLGPEKTSFFQALQIPTKISKGTIEILNEIHLIKKDDRVGASEATLLNMLNI   180
A0A0C5PTI6_AMBCA    VMIPAQNTGLGPEKTSFFQALQIPTKISKGTIEILNEIHLIKKDDRVGASEATLLNMLNI   180
A0A0C9R2E6_AMBAM    VMIPAQNTGLGPEKTSFFQALQIPTKISKGTIEILNEIHLIKKDDRVGASEATLLNMLNI   180
G3MMD6_AMBMA        VMIPAQNTGLGPEKTSFFQALQIPTKISKGTIEILNEIHLIKKDDRVGASEATLLNMLNI   180
A0A023GIB1_AMBTR    VMIPAQNTGLGPXXXSFFQALQIPTKISKGTIEILNEIHLIKKDDRVGASEATLLNMLNI   180
                    * ** *******   ******:*****:********************************

OeRPP0              SPFSYGLKILQVYDSGTVFSPDILDITPEDLRSAFVEGVRNIAAMSLAIGYPTVASVPHS   240
A0A1Z5LG66_ORNMO    SPFSYGLKILQVYDSGTVFSPDILDITPEDLRSAFVEGVRNIAAMSLAIGYPTVASVPHS   240
A0A2R5L843_ORNTU    SPFSYGLKILQVYDSGTVFSPDVLDITPEDLRSAFVDGVRNIASVSLAIGYPTVASVPHS   240
A0A131XRJ4_IXORI    SPFSYGLKILQVYDSGTVFSPEILDITPEDLRSAFVEGVRNVASVSLAIGYPTVASAPHS   240
A0A0K8REE7_IXORI    SPFSYGLKILQVYDSGTVFSPEILDITPEDLRSAFVEGVRNVASVSLAIGYPTVASAPHS   240
B7PRG2_IXOSC        SPFSYGLKILQVYDSGTVFSPEILDITPEDLRSAFVEGVRNVASVSLAIGYPTVASAPHS   240
Q4PMB4_IXOSC        SPFSYGLKILQVYDSGTVFSPEILDITPEDLRSAFVEGVRNVASVSLAIGYPTVASAPHS   240
A0A0C5Q1J9_DERNI    SPFSYGLKILQVYDSGTVFSPDILDITPEDLRSAFVEGVRNVAAVSLSIGYPTVASVPHS   240
A0A131XM65_HYAEX    SPFSYGLKILQVYDSGTVFSPDILDITPEDLRSAFVEGVRNVAAVSLSIGYPTVASVPHS   240
A0A0C5PNS0_RHISA    SPFSYGLKILQVYDSGTVFSPDILDITPEDLRSAFVEGVRNVAAVSLSIGYPTVASVPHS   240
A0A0P0D331_RHIHE    SPFSYGLKILQVYDSGTVFSPDILDITPEDLRSAFVEGVRNVAAVSLSIGYPTVASVPHS   240
A0A224YGD4_RHIZA    SPFSYGLKILQVYDSGTVFSPDILDITPEDLRSAFVEGVRNVAAVSLSIGYPTVASVPHS   240
S5FVY5_RHIMP        SPFSYGLKILQVYDSGTVFSPDILDITPEDLRSAFVEGVRNVAAVSLSIGYPTVASVPHS   240
L7MM43_RHIPU        SPFSYGLKILQVYDSGTVFSPDILDITPEDLRSAFVEGVRNVAAVSLSIGYPTVASVPHS   240
A9Y1V1_HAELO        SPFSYGLKILQVYDSGTVFAPDILDITPEDLRSAFVEGVRNVASVSLAIGYPTVASAPHS   240
A0A023FKN1_AMBCA    SPFSYGLKILQVYDSGTVFSPDILDITPEDLRSAFVEGVRNVASVSLAIGYPTVASAPHS   240
A0A023FIA8_AMBCA    SPFSYGLKILQVYDSGTVFSPDILDITPEDLRSAFIEGVRNVASVSLAIGYPTVASAPHS   240
A0A0C5PTI6_AMBCA    SPFSYGLKILQVYDSGTVFSPDILDITPEDLRSAFVEGVRNVASVSLAIGYPTVASAPHS   240
A0A0C9R2E6_AMBAM    SPFSYGLKILQVYDSGTVFSPDILDITPEDLRSAFVEGVRNVASVSLAIGYPTVASAPHS   240
G3MMD6_AMBMA        SPFSYGLKILQVYDSGTVFSPDILDITPDDLRAAFVEGVRNVASVSLAIGYPTVASVPHS   240
A0A023GIB1_AMBTR    SPFSYGLKILQVYDSGTVFSPDILDITPDDLRAAFVEGVRNVASVSLAIGYPTVASVPHS   240
                    *******************:*::*****:***:**::****:*::**:********.***

OeRPP0              IVNGLKNLIAVAVETDISFPAAEMAKEYLKDPSKFAAVAAAAA-----PAAGGGGA--GQ   293
A0A1Z5LG66_ORNMO    IVNGLKNLIAVAVETDISFPAAEMAKEYLKDPSKFAAVAAAAA-----PAAGGGGA--GQ   293
A0A2R5L843_ORNTU    IVNGLKNLIAVAVETDISFPAAEMAKEYLKDPSKFAAVAAAAA-----PAGGGDKA--GQ   293
A0A131XRJ4_IXORI    IVNGLKNLIAVALETDITFKEAEMAKEYLKDPSKFVAAAAAAA-----PAAGGGAA--AK   293
A0A0K8REE7_IXORI    IVNGLKNLIAVALETDITFKEAEMAKEYLKDPSKFVAAAAAAA-----PAAGGGAA--AK   293
B7PRG2_IXOSC        IVNGLKNLIAVALETDITFKEAEMAKEYLKDPSKFVAAAAAAA-----PAAGGGAA--AK   293
Q4PMB4_IXOSC        IVNGLKNLIAVALETDITFKEAEMAKEYLKDPSKFVAAAAAAA-----PAAGGGAA--AK   293
A0A0C5Q1J9_DERNI    IVNGLKNLIAIAVETDITFKEAEMAKEYLKDPSKFVAAAAP--------AAGGGAAAAKP   292
A0A131XM65_HYAEX    IVNGLKNLIAVAVETDITFKEAEMAKEYLKDPSKFAAAAAP--------AAGGGAAAAKP   292
A0A0C5PNS0_RHISA    IVNGLKNLIAIAVETDITFKEAEMAKEYLKDPSKFAAAAAP--------AAGGGAAAAKP   292
A0A0P0D331_RHIHE    IVNGLKNLIAIAVETDITFKEAEMAKEYLKDPSKFAAAAAP--------AAGGGAAAAKP   292
A0A224YGD4_RHIZA    IVNGLKNLIAIAVETDITFKEAEMAKEYLKDPSKFAAAAAP--------AAGGGAAAAKP   292
S5FVY5_RHIMP        IVNGLKNLIAIAVETDITFKEAEMAKEYLKDPSKFAAAAAP--------AAGGGAAAAKP   292
L7MM43_RHIPU        IVNGLKNLIAIAVETDITFKEAEMAKEYLKDPSKFAAAAAP--------AAGGGAAAAKP   292
A9Y1V1_HAELO        IVNGLKNLIAIALETDITFKEAEMAKEYMKDPSKFAAAAAAAP------AAGGGAAGAKP   294
A0A023FKN1_AMBCA    IVNGLKNLIAIAVETDITFKEAEMAKEYLKDPSKFAAAAAPPPAAGAAAAAGGGAAAAKP   300
A0A023FIA8_AMBCA    IVNGLKNLIAIAVETDITFKEAEMAKEYLKDPSKFAAAAAP--------AAGGGAAAAKP   292
A0A0C5PTI6_AMBCA    IVNGLKNLIAIAVETDITFKEAEMAKEYLKDPSKFAAAAAP--------AAGGGAAAAKP   292
A0A0C9R2E6_AMBAM    IVNGLKNLIAIAVETDITFKEAEMAKEYLKDPSKFAAAAAP--------AAGGGAAAAKP   292
G3MMD6_AMBMA        IVNGLKNLIAIAVETDITFKEAEMAKEYLKDPSKFAAAATP--------AAGGGAAAAKP   292
A0A023GIB1_AMBTR    IVNGLKNLIAIAVETDITFKEAEMAKEYLKDPSKFAAAATP--------AAGGGAAAAKP   292
                    **********:*:****:*  *******:******.*.*:         *.**. *
```

```
                                                                % Identity
OeRPP0              PEAKKEEAKKEESEEEDDDMGFGLFD      319      -
A0A1Z5LG66_ORNMO    PEAKKEEAKKEESEEEDDDMGFGLFD      319      99.4
A0A2R5L843_ORNTU    PEAKKEEAKKEESEEEDDDMGFGLFD      319      95.3
A0A131XRJ4_IXORI    TDAKKEEAKKEESEEEDDDMGFGLFD      319      91.8
A0A0K8REE7_IXORI    PDAKKEEAKKEESEEEDDDMGFGLFD      319      91.8
B7PRG2_IXOSC        PDAKKEEAKKEESEEEDDDMGFGLFD      319      91.5
Q4PMB4_IXOSC        PDAKKEEAKKEESEEEDDDMGFSLFD      319      91.5
A0A0C5Q1J9_DERNI    EASKKEEAKKEESEEEDDDMGF----      314      91.3
A0A131XM65_HYAEX    EESKKEEAKKEESEEEDDDMGFGLFD      318      91.6
A0A0C5PNS0_RHISA    EESKKEEAKKEESEEEDDDMGF----      314      91.6
A0A0P0D331_RHIHE    EESKKEEAKKEESEEEDDDMGFGLFD      318      91.9
A0A224YGD4_RHIZA    EESKKEEAKKEESEEEDDDMGFGLFD      318      91.6
S5FVY5_RHIMP        EESKKEEAKKEESEEEDDDMGFGLFD      318      91.6
L7MM43_RHIPU        EESKKEEAKKEESEEEDDDMGFGLF-      317      91.3
A9Y1V1_HAELO        AEAKKEEAKKEESEEEDDDMGFGLFD      320      90.2
A0A023FKN1_AMBCA    EEAKKEEAKKEESEEEDDDMGFGLFD      326      91.6
A0A023FIA8_AMBCA    EEAKKEEAKKEESEEEDDDMGFGLFD      318      91.0
A0A0C5PTI6_AMBCA    EEAKKEEAKKEESEEEDDDMGFGLFD      318      91.3
A0A0C9R2E6_AMBAM    EEAKKEEAKKEESEEEDDDMGFGLFD      318      91.2
G3MMD6_AMBMA        EEAKKEEAKKEESEEEDDDMGFGLFD      318      90.0
A0A023GIB1_AMBTR    EEAKKEEAKKEESEEEDDDMGFGLFD      318      91.0
                      :*******************
```

b

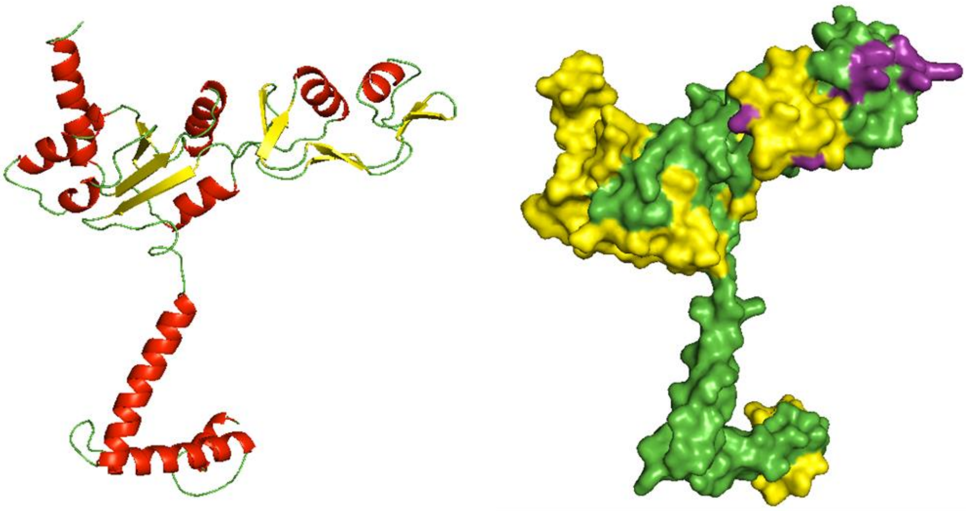

Supplement: Supplementary file 4 — Additional file 4: Figure S3. Tick RPP0 sequence alignment and OeRPP0 topology prediction. [file 13071_2019_3768_MOESM4_ESM.pdf]
