# Supplementary material for: In silico selection of functionally important proteins from the mialome of Ornithodoros erraticus ticks and assessment of their protective efficacy as vaccine targets
Source: Parasit Vectors. 2019 Oct 30;12:508. doi: 10.1186/s13071-019-3768-1 (PMC6822432; doi:10.1186/s13071-019-3768-1)
Supplement: Supplementary file 5 — Additional file 5: Figure S4. Alignment of the amino acid sequences of A0A293MVU8_ORNER (OePK4) and orthologous peptides and proteins. [file 13071_2019_3768_MOESM5_ESM.pdf]

Additional file 5: Figure S4. Uniprot entry names are shown: IXORI, *Ixodes ricinus*; IXOSC, *I. scapularis*; ORNER, *Ornithodoros erraticus*; ORNMO, *O. moubata*; ORNTU, *O. turicata*. The amino acids conserved in all sequences are labelled with asterisks (*); the conservative and semi-conservative substitutions are labelled with colons (:) and periods (.), respectively. The signal peptides are highlighted in blue. The percentage of sequence identity of A0A293MVU8 and the peptide/proteins in the alignment is indicated in the column on the lower right corner.

```
A0A293MVU8_ORNER   MAFKILLCIFATLVVLSLVSARPTE---SESEAEQSEPANEGNAKVKADIEVVGTEVKAK   57
A0A1Z5KWW5_ORNMO   MTSSTLICVFALFAVV---TLTSAQFGRPNFNFKPPAPRHRFG-YGIQAGGNSGKHYGVN   56
Q6QVL8_ORNMO       MTSSTLSCVFALFAVV---TLTSAQFGRPNFNFKPPAPRHRFG-YGIQAGGNSGKHYEVN   56
A0A2R5LAY5_ORNTU   MTSKTLLCIFALLAVV---TLASAQFGRPKFNFQPPPPRHRIE-IAAQAGGKSGKHYNVN   56
A0A1Z5L697_ORNMO   MASKCVVCVLVLLAVAAVCSAQSRSGGRPNFNFRPQPPRHRFE-VFGSGSGRNGRNFNAN   59
A0A131Y9K2_IXORI   MSRVALCLF--VALAVLAIT---HAQRRPNFNFQKPPPRHRFE-LQGSGGGRSRGNYNVN   54
V5HGP9_IXORI       MSRVALCLF--VALAVLAIT---HAQRRPNFNFQKPPPRHRFE-LQGSGGGRNRGNYNVN   54
B7P261_IXOSC       MSRVALYFF--VALAVLAIT---NAQQRPNFNFQKPPPRHRFE-LQGSGGGRNRGNYNVN   54
Q4PMD7_IXOSC       MSRVALYFL--VALAVLAIT---NAQRRPNFNFQKPPPRHRFE-LQGSGGGRNRGNYNVN   54
B7PVH8_IXOSC       -IFLCLVCLVLCTLIFLFCK---LQQRRPNFNFQKPPPRHRFE-LQGSGGGRNRGNYHVN   55
B7PUK6_IXOSC       MSRIALYLFYYVCLIFFFCK---LQQRIPNFNFQQPPPRHRFE-LQGSGGGRNRGNYHVN   56
                        :  .          .         : : .   * :.              .  .:

                                                                               % Identity
A0A293MVU8_ORNER   VEAFLQGDLYRFENGGKVSGHVKGSQSFSSKDGGTW-GSPQVEAGVEVEIPLQ---  109     -
A0A1Z5KWW5_ORNMO   VGARAEYDLHRFPNGAKIVGHVEGSHAFGKYEGQKWQGKPQGEVGVRVEIPFKG--  110    35.5
Q6QVL8_ORNMO       VGARAEYDLHRFPNGAKIVGHVEGSHAFGKYEGQKWQGKPQGEVGVRVEIPFKG--  110    35.5
A0A2R5LAY5_ORNTU   VGGRAEYDLHRFKNGAKIVAHVEGSHSFGKHEGHKWQEKPKGEVGVR---------  103    33.3
A0A1Z5L697_ORNMO   VGARGEYDIHRFKNGGRITGHVEGSQSFGRFNGQSYRGKPQGEVGIRAEIPF----  111    35.0
A0A131Y9K2_IXORI   LGARGEYDIHRFKNGGKVVGYGQGSQSFGRFNGQSYKGKPQGEVGVRVEIPIGKGK  110    34.2
V5HGP9_IXORI       LGARGEYDIHRFKNGGKVVGYGQGSQSFGRFNGQSYKGKPQGEVGVRVEIPIGKGK  110    34.2
B7P261_IXOSC       LGARGEYDIHRFKNGGKVVGYGQGSQSFGRFDGQSYKGKPQGEVGVRVEIPIGKGK  110    34.2
Q4PMD7_IXOSC       LGARGEYDIHRFKNGGKVVGYGQGSQSFGRFDGQSYKGKPQGEVGVRVEIPIGKGK  110    57.8
B7PVH8_IXOSC       LGARGEYDIHRFKNGGKVVGYGQGSQSFGRFDGQSYKGKPQGEVGVRVEIPIGKGK  111    57.8
B7PUK6_IXOSC       LGARGEYDIHRFKNGGKVVGYGQGSQSFGRFDGQSYKGKPQGEVGVRVEIPIGKG-  111    57.8
                   : .  : *::** **.:: .: :**::*.  :* .:  .*: *.*:.
```
