# Supplementary material for: In silico selection of functionally important proteins from the mialome of Ornithodoros erraticus ticks and assessment of their protective efficacy as vaccine targets
Source: Parasit Vectors. 2019 Oct 30;12:508. doi: 10.1186/s13071-019-3768-1 (PMC6822432; doi:10.1186/s13071-019-3768-1)

Additional file 6: Figure S5. Expression and purification of fusion proteins CHI-GST (**a**) and TSP1-GST (**b**), and recombinants TSP2 (**c**), RPP0 (**d**) and PK4 (**e**). Coomassie blue-stained SDS-PAGE gels showing the whole cell lysates before induction (-IPTG); the supernatants (S), and the pellets (P) of the cell lysate after the induction of protein expression with IPTG (+IPTG); the supernatants after pellet solubilisation with 8M urea (+ Urea), and the purified proteins either by electro elution from gel (CHI-GST, TSP1-GST) or by nickel affinity chromatography (PK4, RPP0, TSP2) (arrows). MW. Molecular weight standards in kDa

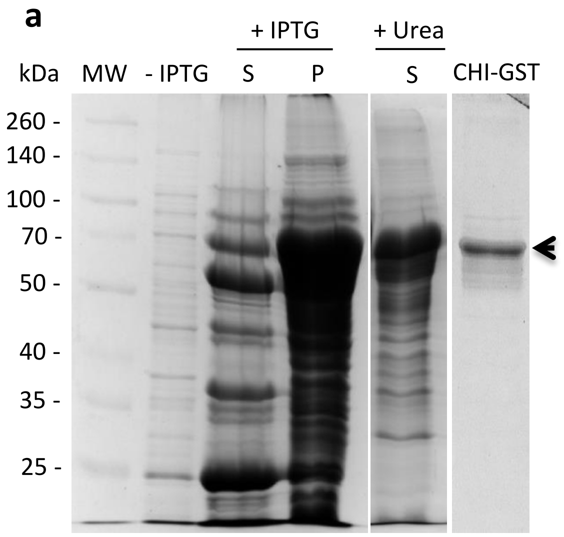

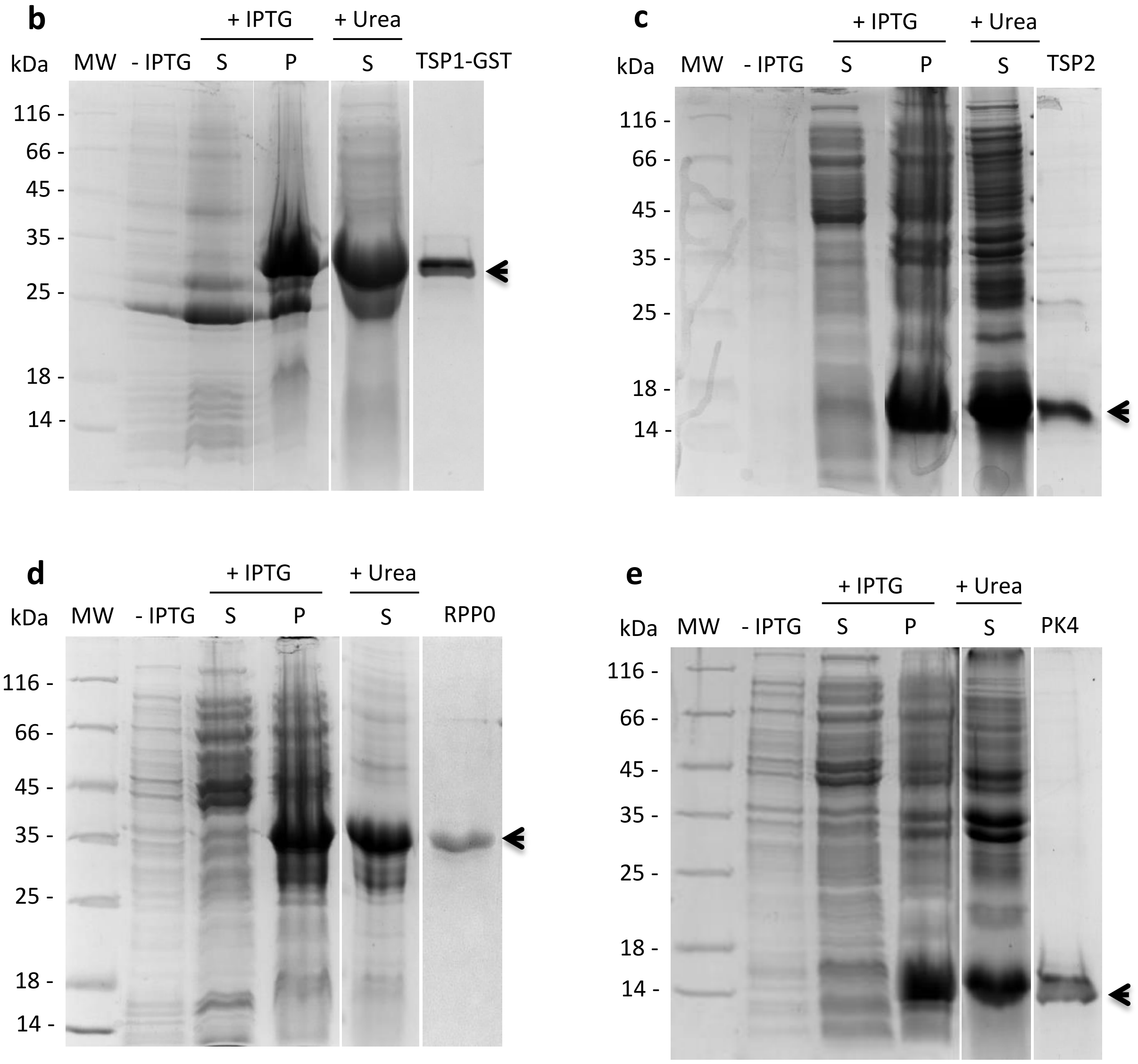

Supplement: Supplementary file 6 — Additional file 6: Figure S5. Obtaining of candidate antigens as recombinant proteins. [file 13071_2019_3768_MOESM6_ESM.pdf]
