# Supplementary material for: In silico selection of functionally important proteins from the mialome of Ornithodoros erraticus ticks and assessment of their protective efficacy as vaccine targets
Source: Parasit Vectors. 2019 Oct 30;12:508. doi: 10.1186/s13071-019-3768-1 (PMC6822432; doi:10.1186/s13071-019-3768-1)

Additional file 7: Figure S6. IgG antibody response in rabbits vaccinated with recombinant antigens OeCHI, OePK4, OeRPP0 and OeTSP1 + OeTSP2 (TSPs) against midgut protein extracts from *Ornithodoros erraticus* (**a**) and *O. moubata* (**b**) female ticks taken either before blood feeding (Unfed) or 48 h post-feeding (Fed). Protein extracts were fractionated into soluble proteins (Sol) and membrane-associated proteins (Mem). Values are the average OD ± SD at 492 nm ($OD_{492\ nm}$ immune sera – $OD_{492\ nm}$ preimmune sera) from each rabbit group. Sera were taken before immunisation (preimmune), 14 days post-immunisation (14 d.p.i.), immediately before the infestation and 14 days post-infestation (28 d.p.i.) and were used at 1/300 dilution.

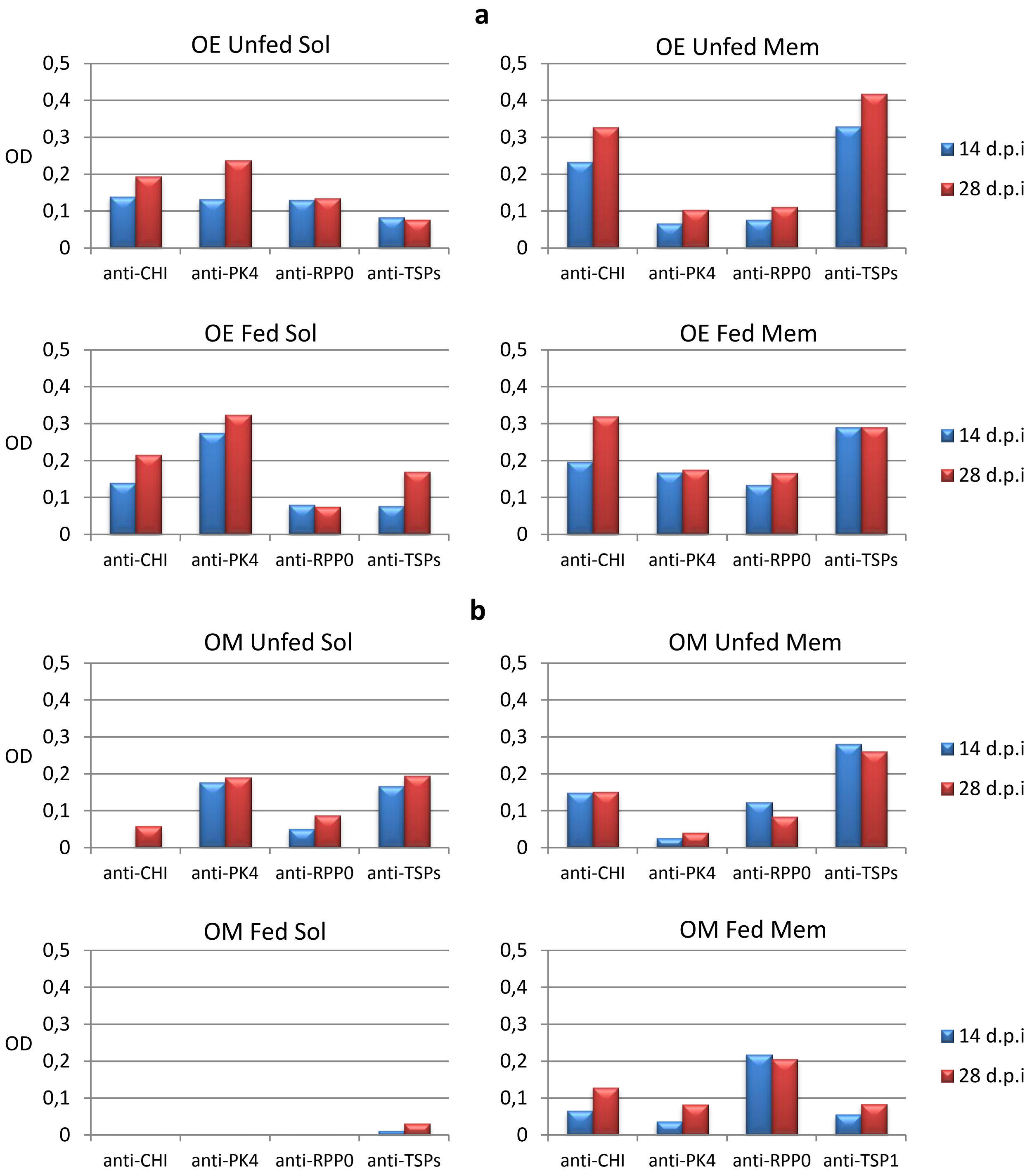

Supplement: Supplementary file 7 — Additional file 7: Figure S6. Analysis of reactivity of the vaccinated rabbit sera by ELISA. [file 13071_2019_3768_MOESM7_ESM.pdf]
